# Supplementary material for: Privacy-preserving multicenter differential protein abundance analysis with FedProt
Source: Nat Comput Sci. 2025 Jul 11;5(8):675–88. doi: 10.1038/s43588-025-00832-7 (PMC12374843; doi:10.1038/s43588-025-00832-7)
Supplement: Supplementary file 1 — Supplementary results, methods, Figs. 1–20 and Tables 1–6 and 8–13. [file 43588_2025_832_MOESM1_ESM.pdf]

nature computational science

Supplementary information https://doi.org/10.1038/s43588-025-00832-7

Privacy-preserving multicenter differential protein abundance analysis with FedProt

In the format provided by the authors and unedited

Supplementary Information

Supplementary Results..2
Handling of batch effects..2
Integrating quantification tables from different software..3
Integrating diverse studies in the absence of raw data...4
Impact of varying thresholds on the performance of decentralized methods.....................5
Impact of varying sample sizes on the performance of decentralized methods.................6
Supplementary Figures..7
Supplementary Tables..23
Supplementary Methods..33
Meta-analysis approaches..33
Human serum dataset – LC-MS/MS measurement..34
Sample preparation..34
Sample preparation for mass spectrometry..34
LC-MS/MS measurement..34
Bacterial dataset – LC-MS/MS measurement..37
Sample preparation..37
Sample preparation for mass spectrometry..37
LC-MS/MS measurement..39
Data exchange using the FeatureCloud infrastructure..42
Design mask creation (FedProt step)..43
Supplementary References..45

Supplementary Results

Handling of batch effects

Batch effects are a critical issue in data analysis since they can mask true biological differences and lead to incorrect conclusions. It is crucial to handle batch effects properly to ensure the validity of differential abundance analysis results. For both bacterial and human serum datasets, principal component analysis revealed large differences between samples from different cohorts, exceeding differences within the same cohort (**Supplementary Figure 11**). This lab-specific batch effect was particularly noticeable when comparing samples from labs A and B, who performed cell lysis, and those from labs C, D, and E, who worked with centrally prepared cell lysates.

Since unaccounted lab-specific batch effects can severely confound the analysis results[1], researchers either adjust the data to remove batch effects before the analysis or modify the model to account for them. *ComBat*[2], a popular batch effect correction tool, does not accept data with missing values, making it unsuitable for multicenter proteomic data, where the number of rows with missing values increases with the number of participating laboratories. As imputation reliability is questionable, the recent HarmonizR workflow has been developed to handle inputs with missing values[3]. However, the current version of HarmonizR cannot account for confounders, which is necessary to avoid overcorrection in imbalanced data.

To ensure FedProt's approach to handling differential expression analysis and batch effect adjustment is not inferior to popular approaches involving direct data adjustment, we compared its results with those of DEqMS applied to pooled data after batch effect correction using the *removeBatchEffect* function from the limma R package[4], which is able to account for covariates and correct the data with missing values. Similar to Flimma[5] and the limma R package, to account for batch effects in the analysis involving m clients, it selects one client that serves as the reference batch and adds to the design matrix $m-1$ binary covariates modeling expression changes of each client w.r.t. the reference batch.

Including batch factors in the design is recommended to correctly assess standard errors[4]. Using pre-corrected data as "batch effect free" in the analysis may lead to exaggerated confidence[6]. We observed these effects on adjusted p-value results by comparing two DEqMS analyses on centrally aggregated data: one corrected using limma's *removeBatchEffect* function and the other on uncorrected data with batch factors included in the model (**Supplementary Figure 13**).

Nevertheless, we can compare log-fold changes to evaluate FedProt against central analysis on batch effect corrected data and thus visualize removing the batch effect. For both datasets, log fold-changes were perfectly correlated (r=1, rho=1) between FedProt on non-corrected data and centralized *DEqMS* workflow on corrected data with maximal absolute differences no greater than 5.5E-14 (**Supplementary Figure 14**).

Integrating quantification tables from different software

To evaluate FedProt performance in integrating heterogeneous quantification tables, we used the bacterial dataset that was preprocessed using different quantification software across multiple centers (see **Supplementary Table 4**). So, not only were the LC-MS set-ups different between centers, but also raw data preprocessing (quantification software run) was done in different research centers on different machines.

The results showed that different tools and tools' versions used for quantification may lead to differences in protein identification. Those may complicate the optimal intersection of protein groups across centers. As shown in **Supplementary Figure 15**, the overall number of protein groups available for analysis is not negatively impacted by these preprocessing differences. As suggested in the DIA-NN documentation[7], the intersection of protein groups in DIA data across centers can be improved by emulating match-between-runs (MBR): first creating a spectral library in one center and then using it to analyze data from other centers. When raw data reanalysis is not possible and only quantification tables are available, collapsing protein groups to gene names may help[8], although this may result in the loss of isoform-specific details. In general, FedProt is designed to integrate protein group (PG × sample) matrices across centers regardless of the quantification software used.

Our results using non-uniform raw data quantification across centers indicate that while FedProt can accommodate slight variations in preprocessing, but overall the performance in such scenarios is comparable to that obtained by the best meta-analysis approaches (see **Supplementary Figure 10, panels a-c, and Supplementary Table 8**). At the same time, if we compare it with the results of centralized DEqMS on non-uniformly quantified data, we can see that FedProt produces the same results as the central analysis with negligibly small deviations (**Supplementary Figure 10, panels d-f, and Supplementary Table 8**). Thus, if raw files are available, it is better to preprocess them as uniformly as possible. If there is no access to raw data, FedProt shows the same result as central analysis on the same data, outperforming meta-analyses.

Quantification in Lab A and Lab E was performed using Spectronaut 17 in directDIA+ mode with a 1% FDR, and data preprocessing included Q.value filtering with a threshold of 0.01. The parameters for DIA-NN have been described in detail in the main methods section.

Integrating diverse studies in the absence of raw data

FedProt is designed to accommodate variability in sample preparation and raw data acquisition, as demonstrated by our analyses of bacterial and human serum datasets. Consequently, FedProt is well-suited for combining datasets from different studies, including those available in public repositories such as PRIDE. Although optimal integration is achieved when quantification software and preprocessing protocols are uniform, FedProt can address challenges posed by heterogeneous preprocessing, provided that the central analysis remains biologically meaningful. The success of the integration also depends on the compatibility of the underlying biological assumptions across datasets.

To illustrate the robustness of FedProt in handling heterogeneous data, we analyzed publicly available proteomics datasets for clear-cell renal cell carcinoma (ccRCC). Three datasets were used in this analysis: PDC000127[9], PXD042844[10], PXD030344[11] (**Supplementary Table 5**).

The published proteomics data matrices were utilized, with gene names serving as the common identifier across datasets. Peptide count information was not used. The PXD042844 and PXD030344 datasets were median normalized and log-transformed, while the PDC000127 dataset was used as provided online. Data filtering was applied to retain only rows with at least one value per target class. Principal component analysis plots and the intersection of feature (gene) names are provided in **Supplementary Figure 16**.

To evaluate FedProt and meta-analyses, differential abundance analysis was performed between tumor and control samples using the processed data. The same data tables, pooled, were centrally analyzed using the DEqMS method to establish the ground truth.

Our analysis confirmed that FedProt applied to the ccRCC datasets preprocessed differently again yielded results the same as centralized analyses (see **Supplementary Figure 17**). Specifically, the maximum absolute differences between FedProt results and those from the centralized DEqMS analysis are no greater than 8×10^{-13} for both log2FC and negative log-transformed adjusted p-values. At the same time, however, the best meta-analysis performance in terms of Jaccard similarity coefficient was 0.86 (**Supplementary Table 9**).

The analysis illustrates that FedProt can effectively integrate data from different studies, even when sample preparation and data preprocessing protocols vary. This capability underscores the potential utility of FedProt in studies where sample sizes from single centers may be limited, such as understudied, orphan or rare disease studies, particularly when raw data is unavailable and uniform *in silico* preprocessing cannot be performed.

Impact of varying thresholds on the performance of decentralized methods

We evaluate the impact of varying log2FC and adj. p-value cutoffs on the performance of FedProt and meta-analysis methods. To do so, we used the bacterial and the human serum datasets and compared a range of cutoffs (0.15-2.75 for log2FC and 0.001-0.05 for adj.p-value). We tested how these thresholds influenced the number of identified differentially abundant proteins and key performance metrics, including error rates (false positives and false negatives) and Jaccard similarity coefficients relative to centralized analysis results.

In practical applications, the selection of log2FC and adj. p-value thresholds depend on many factors, including the experimental system, specific phenotype, and research objectives. Although in many studies researchers choose cutoffs |log2FC|>2 or |log2FC|>1, in the human serum dataset with a small effect size for proteins, setting a logFC cutoff of 1 resulted in no detected true positives, meaning that no differentially abundant proteins were identified even in the centralized scenario. In the bacterial dataset, only 2.64% of all measured proteins were significantly (adj.p-value < 0.05) differentially abundant with |log2FC|>1 (**Supplementary Figure 6**).

The analyses showed that in both datasets, regardless of the chosen thresholds, FedProt results always remained the same as the results of centralized analyses. Meta-analyses results, in contrast, tended to diverge more from the results of centralized analyses when the logFC threshold was relaxed, especially for smaller log2FC cutoffs (**Supplementary Figures 7** and **8**). The discrepancies between meta-analysis methods and centralized analysis results are higher when focusing on smaller effects, as in the human serum dataset.

The result highlights the importance of selecting threshold values that reflect realistic biological effect sizes for each dataset type and also demonstrates that FedProt provides results identical to the central analysis regardless of the specific choice of |log2FC| and adj. p-value cutoffs.

Impact of varying sample sizes on the performance of decentralized methods

Modern MS-based proteomics cohort designs are shifting toward larger sample sizes, typically several hundred to a few thousand samples. However, large-scale multi-center MS-based datasets still remain relatively uncommon compared to antibody-based platforms such as Olink[12]. Most available MS-based datasets include only hundreds to low thousands of samples, with studies (especially multi-center or ring trials) often limited to a few hundred samples per cohort. In this context, FedProt is designed to help researchers increase cohort size while preserving data privacy, addressing a key limitation in current MS-based proteomics studies.

One of the main components of FedProt is federated linear regression[13], which is designed to exactly replicate the results of a centralized analysis regardless of sample size. To illustrate this, we analyzed FedProt and meta-analyses performance using simulated data with 66, 660, and 6600 samples (see **Supplementary Table 10**). We maintained a constant set of 6000 proteins in each simulation with approximately 350 differentially abundant proteins. According to the simulation method, the exact number of differentially abundant proteins may vary slightly randomly. The results (**Supplementary Figure 18 and Supplementary Table 11**) demonstrate that FedProt's results remain consistent with those from centralized analysis, even as the cohort size increases.

The analyses indicate that meta-analyses improve with increased sample size. In smaller cohorts, meta-analysis methods produced results that diverged from the centralized analysis, regardless of whether the dataset was balanced or imbalanced. With a large sample size (6600 samples), meta-analyses could accurately rank the top 350 proteins (**Supplementary Figure 18**). However, when considering all proteins and applying both log fold-change and p-value simultaneously (**Supplementary Table 11**), meta-analyses still exhibited discrepancies compared to the central analysis, with these differences being more pronounced in imbalanced datasets.

These experiments demonstrate that FedProt scales effectively and maintains stable performance across varying cohort sizes. In contrast, meta-analyses do not fully replicate centralized results in more complex data scenarios. This indicates that the gain from running a federated analysis over a standard meta-analysis remains substantial, even with large sample sizes, achieving consistency with centralized results and ensuring robust performance under diverse conditions.

Supplementary Figures

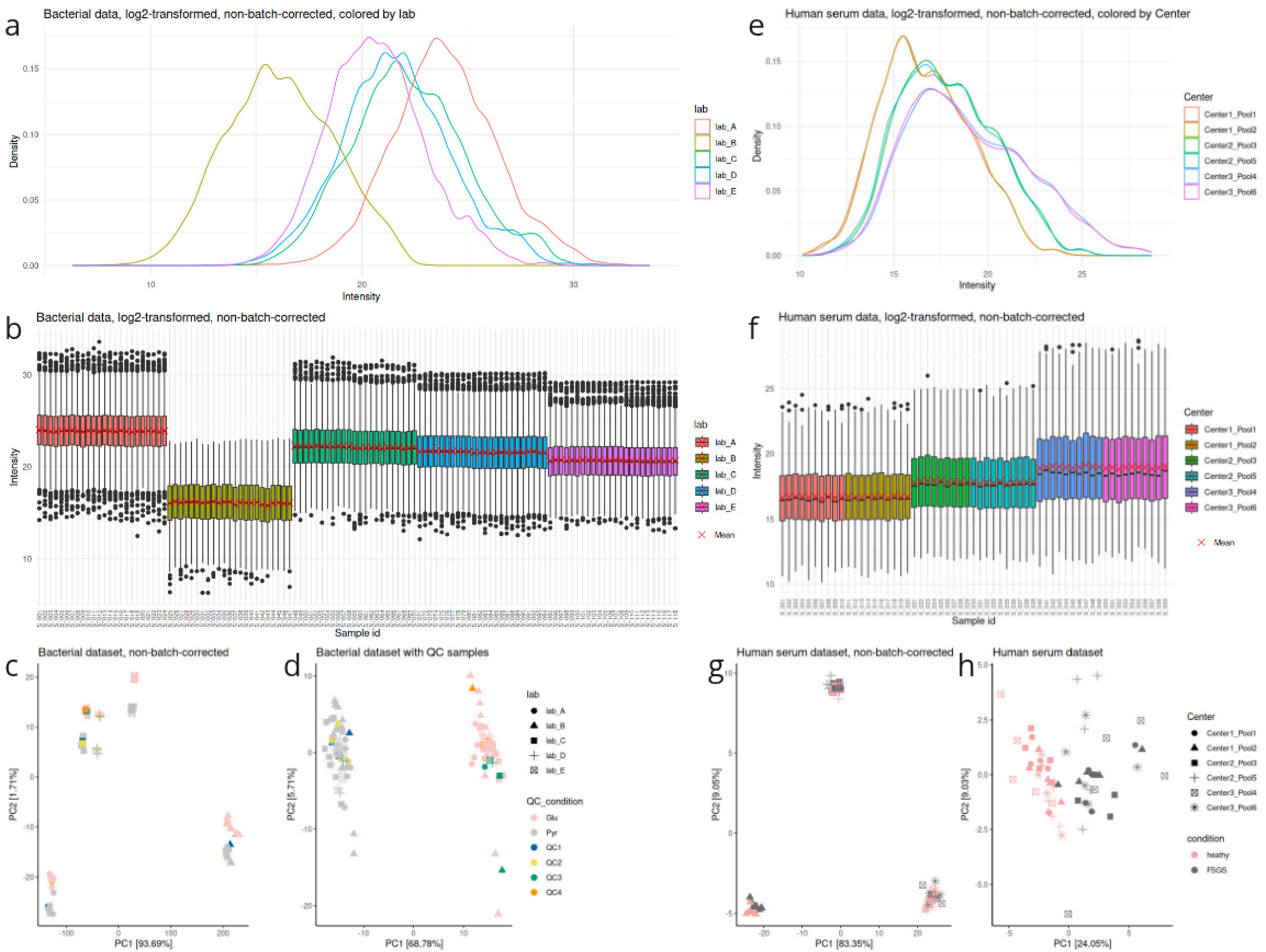
a
Bacterial data, log2-transformed, non-batch-corrected, colored by lab
Density
Intensity
lab
lab_A
lab_B
lab_C
lab_D
lab_E
b
Bacterial data, log2-transformed, non-batch-corrected
Intensity
Sample id
lab
lab_A
lab_B
lab_C
lab_D
lab_E
Mean
c
Bacterial dataset, non-batch-corrected
PC1 [93.69%]
PC2 [1.71%]
d
Bacterial dataset with QC samples
PC1 [68.78%]
PC2 [5.71%]
lab
lab_A
lab_B
lab_C
lab_D
lab_E
QC_condition
Glu
Pyr
QC1
QC2
QC3
QC4
e
Human serum data, log2-transformed, non-batch-corrected, colored by Center
Density
Intensity
Center
Center1_Pool1
Center1_Pool2
Center2_Pool3
Center2_Pool5
Center3_Pool4
Center3_Pool6
f
Human serum data, log2-transformed, non-batch-corrected
Intensity
Sample id
Center
Center1_Pool1
Center1_Pool2
Center2_Pool3
Center2_Pool5
Center3_Pool4
Center3_Pool6
Mean
g
Human serum dataset, non-batch-corrected
PC1 [83.35%]
PC2 [9.05%]
h
Human serum dataset
PC1 [24.05%]
PC2 [9.03%]
Center
Center1_Pool1
Center1_Pool2
Center2_Pool3
Center2_Pool5
Center3_Pool4
Center3_Pool6
condition
heathy
FSGS

Supplementary Figure 1. Descriptive statistics and principal component analysis (PCA) plots for the bacterial (panels a-d) and human serum (panels e-h) datasets.

Intensity density plots (panels a, e) are shown for log2-transformed data, colored by the lab for the bacterial dataset (panels a and b) and by centers and TMT pools for the human serum dataset (panels e and f). PCA plots before (as used in FedProt, panels c and g) and after batch effect correction (panels d and h) using the limma removeBatchEffect function for the bacterial dataset, including QC samples (panels c and d) and for the human serum dataset (panels g and h). QC samples in the bacterial dataset represent technical replicates generated for QC purposes and removed from the dataset during FedProt evaluation. Panels b and f: boxes show the inter-quartile range (25th-75th percentile); the horizontal line denotes the median (50th percentile) and the red cross denotes the mean. Whiskers extend to the furthest data point ≤ 1.5 × IQR from each hinge; points beyond this limit are plotted individually as outliers.

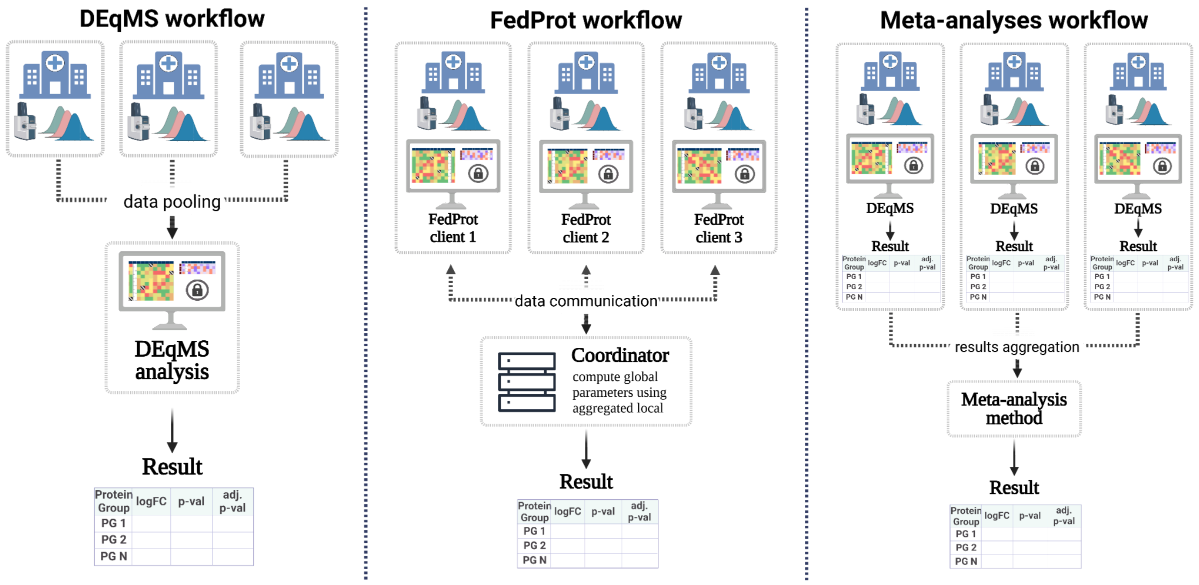

Supplementary Figure 2. Protein abundance analysis workflows in case of multi-center studies. Dashed areas highlight different physical locations.

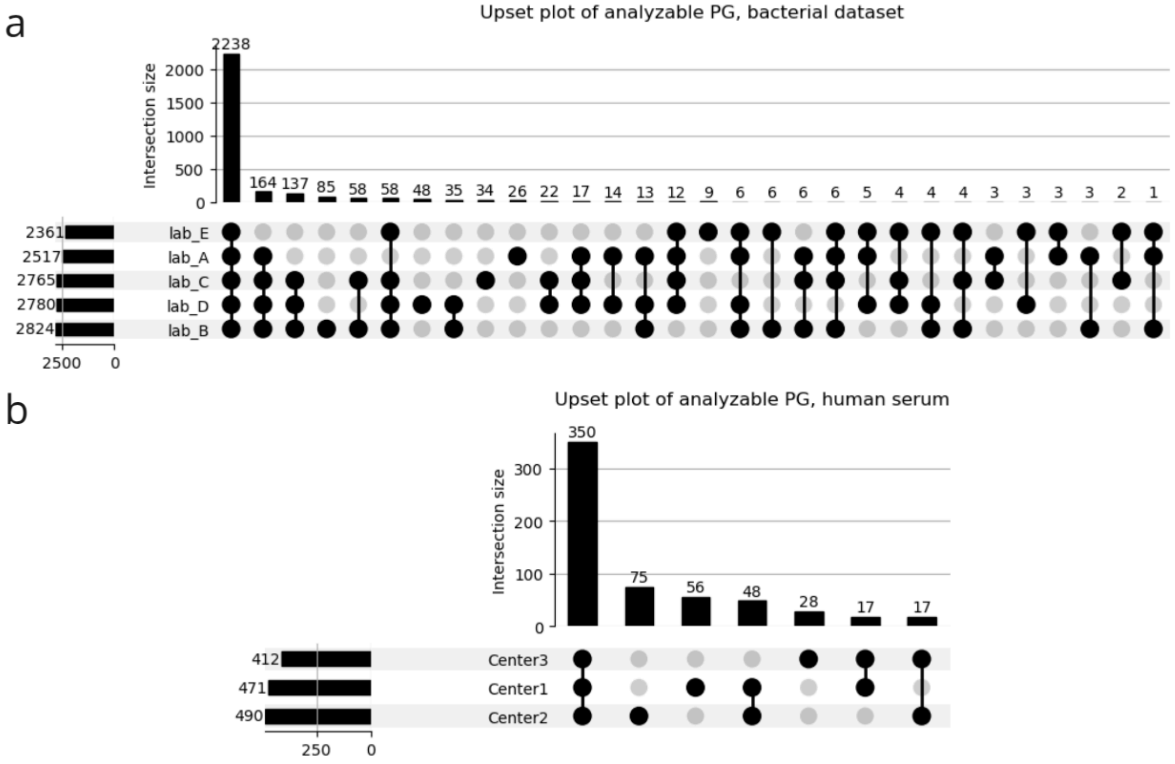

Supplementary Figure 3. The number of protein groups that could be analyzed by the DEqMS method inside each lab separately.

Panel a — for the bacterial dataset; b — for the human serum dataset.

a

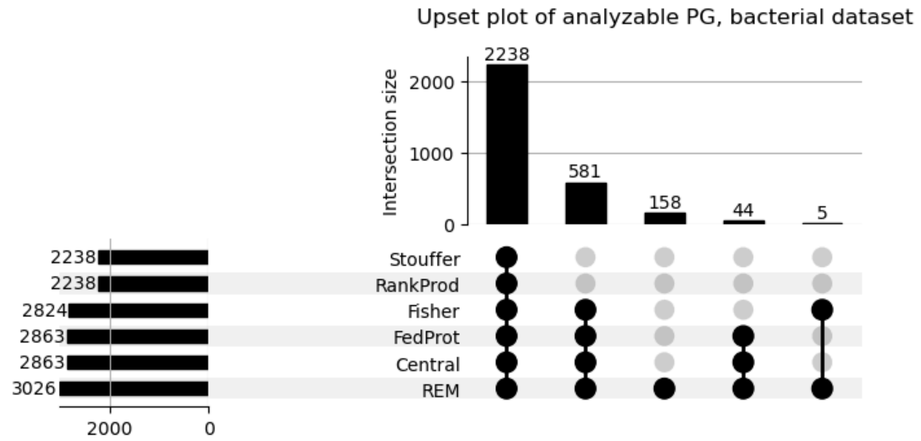

a

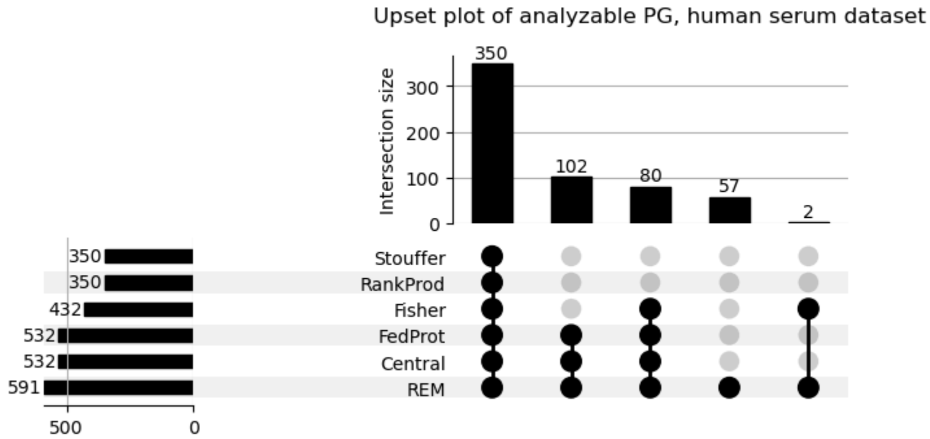

Supplementary Figure 4. The number of protein groups (PG) that could be analyzed by the central DEqMS method, FedProt, and meta-analysis methods.
Panel a — for the bacterial dataset; b— for the human serum dataset.

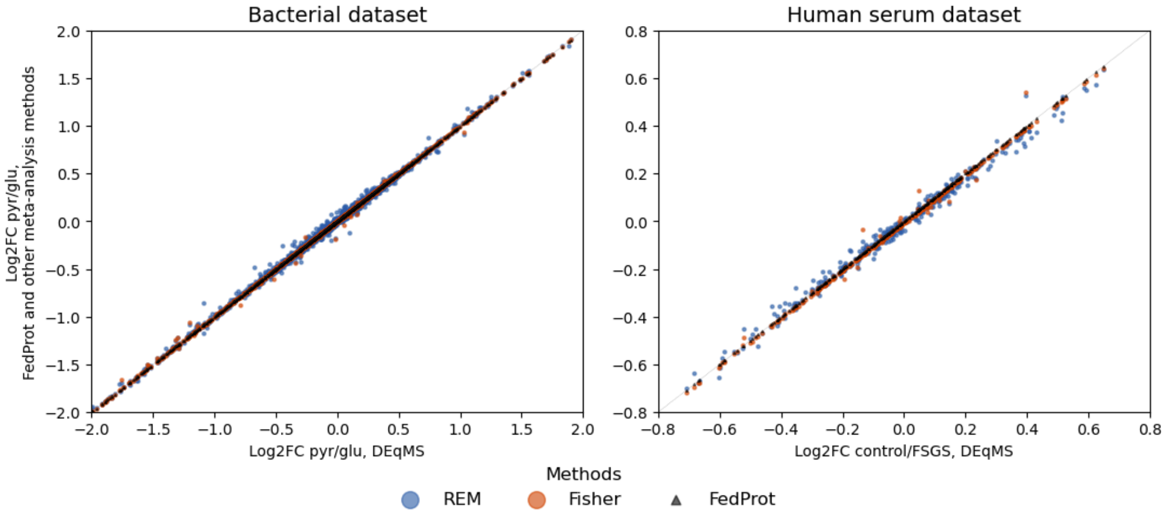

Supplementary Figure 5. The comparison of log-fold changes computed by FedProt or meta-analysis methods (y-axis) with centralized analysis (x-axis).

For the bacterial dataset, only values falling within the interval [-2,2] are shown; of the entire dataset, 3% of the values do not fall within this interval. For the human serum dataset, only values falling within the interval [-0.8,0.8] are shown; of the entire dataset, 0.8% of the values do not fall within this interval.

The thin black line is the diagonal.

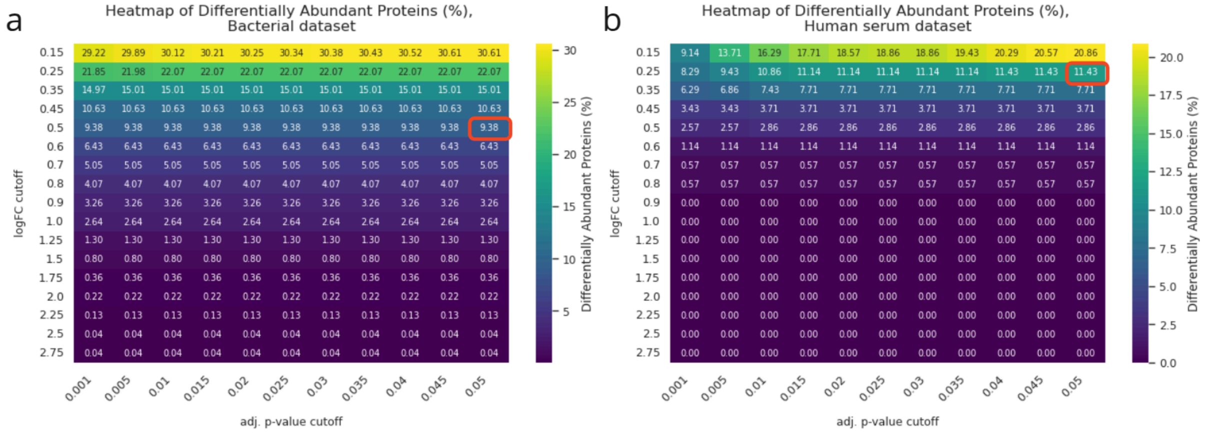

Supplementary Figure 6. The proportion of differentially abundant proteins in centralized analysis (DEqMS) under different adj.p-value and logFC cutoffs.

Panel a shows the bacterial dataset, panel b – human serum dataset results. The red square marks the selected cutoff for each dataset. The number of protein groups analyzed for the bacterial dataset is n=2238 and for the human serum dataset is n=350. The exact results (with p-values) are uploaded to the Zenodo[14].

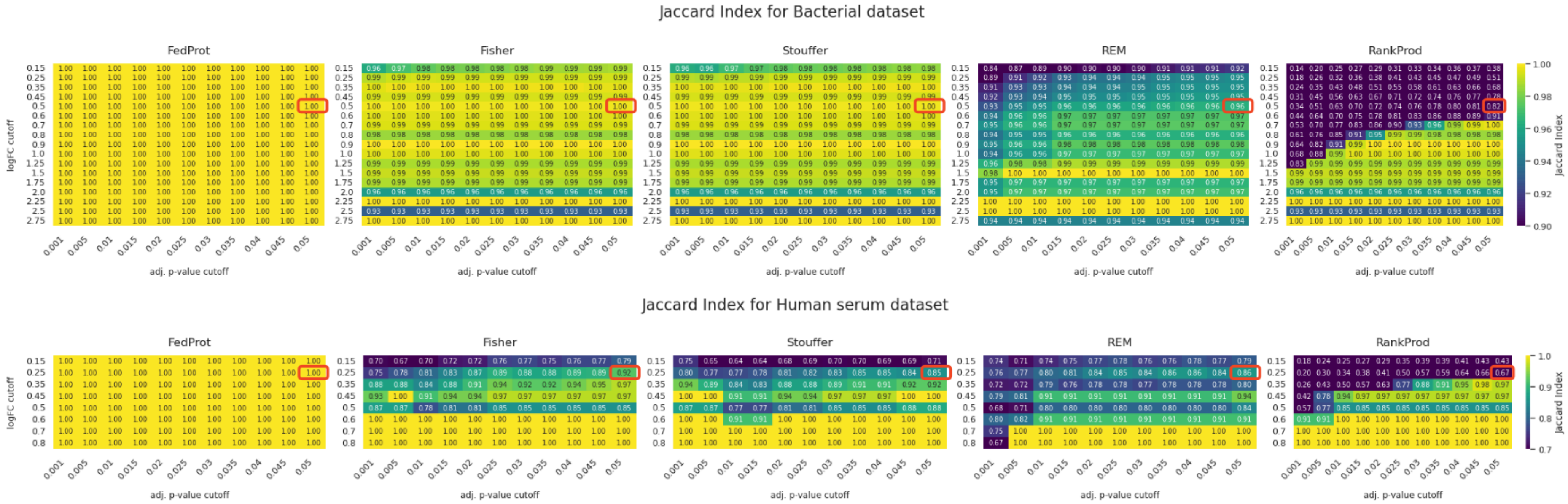

Supplementary Figure 7. Consistency between centralized and decentralized methods for the bacterial and human serum datasets in terms of Jaccard index for different adj.p-value and logFC cutoffs.

The red square marks the logFC and adj.p-value cutoff selected for the main analysis.The number of protein groups analyzed for the bacterial dataset is n=2238 and for the human serum dataset is n=350. The exact results (with p-values) are uploaded to the Zenodo[14].

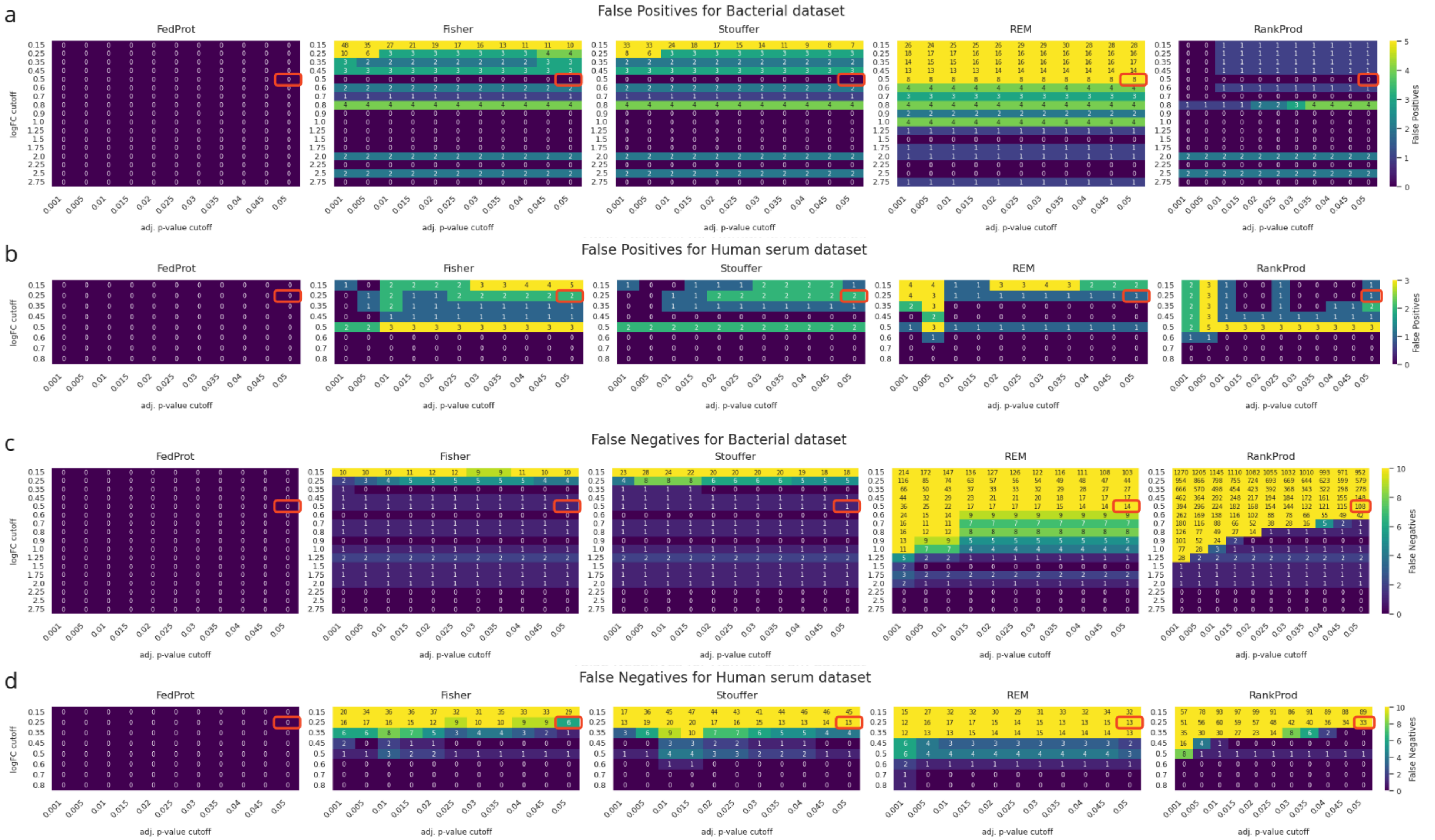

Supplementary Figure 8. Error rates for the results of FedProt and selected meta-analysis approaches compared to centralized DEqMS results using bacterial and human serum dataset.

Error rates (false positives – panels a, b; and false negatives – panels c, d) for the bacterial (panels a, c) and human serum (panels b, d) datasets for different adj.p-value and logFC cutoffs. The red square marks the logFC and adj.p-value cutoff selected for the main analysis.

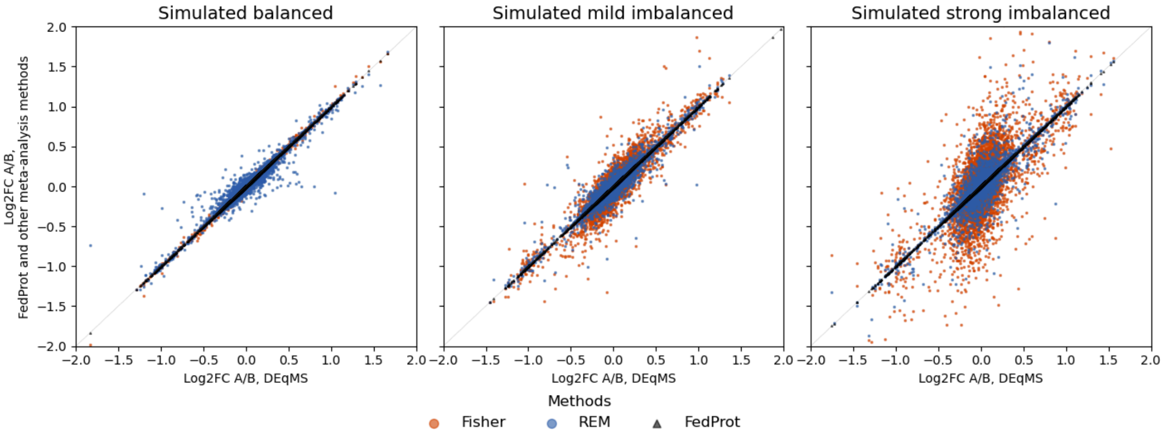

Supplementary Figure 9. The comparison of log-fold changes computed by FedProt or meta-analysis methods (y-axis) with centralized analysis (x-axis) for one out of 50 analysis runs for each scenario.

For the simulated datasets, only values falling within the interval [-2,2] are shown. For mild imbalanced dataset, only 0.1% of values do not fall within this interval; for strong imbalanced – 0.2%.

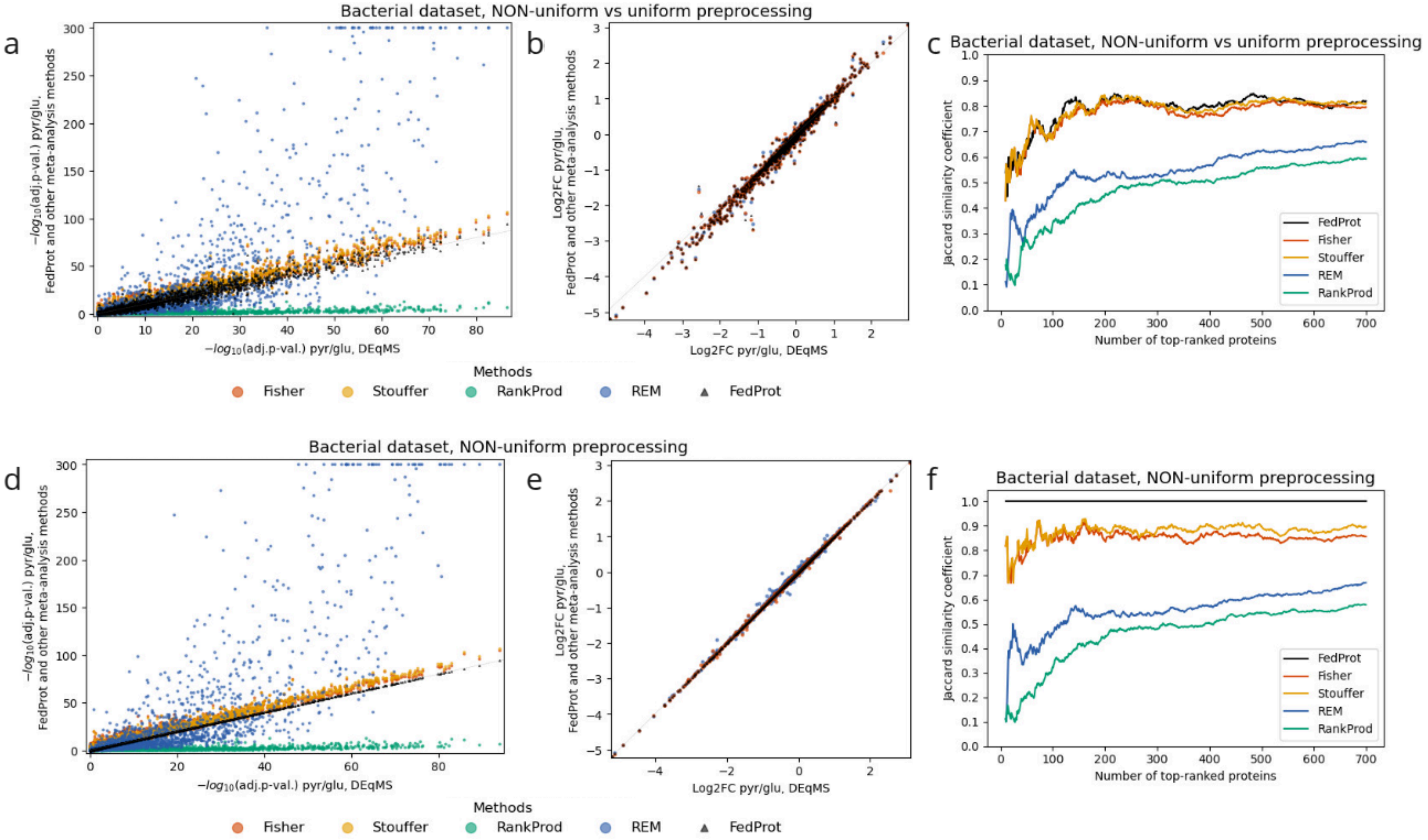

Supplementary Figure 10. **The performance of FedProt and meta-analyses using non-uniformly preprocessed data compared to results of central DEqMS analysis with uniformly preprocessed data (panels a-c) and non-uniformly preprocessed data (panels d-f).**

Panels a, b and d, e: The comparison of negative log-transformed adjusted p-values (panels a, d) and logFC (panels b, e) computed by FedProt or meta-analysis methods (y-axis, non-uniform preprocessing) with the centralized DEqMS analysis (x-axis, uniform preprocessing). The thin gray line is the diagonal.

Panels c and f: The dependency of the Jaccard similarity coefficient on the number of top-ranked proteins identified by the centralized DEqMS and decentralized approaches, proteins were ranked based on their decreasing negative log-transformed BH-adjusted p-values and not filtered by log2FC.

Metrics use each method’s own |log2FC| and BH-adjusted p-values. The number of protein groups analyzed here is n=2304. The exact results (with p-values) are uploaded to the Zenodo[14].

a

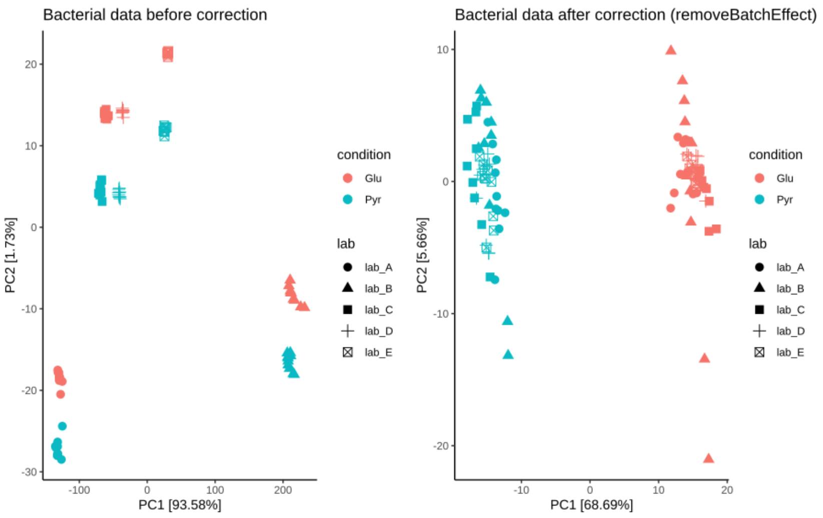

b

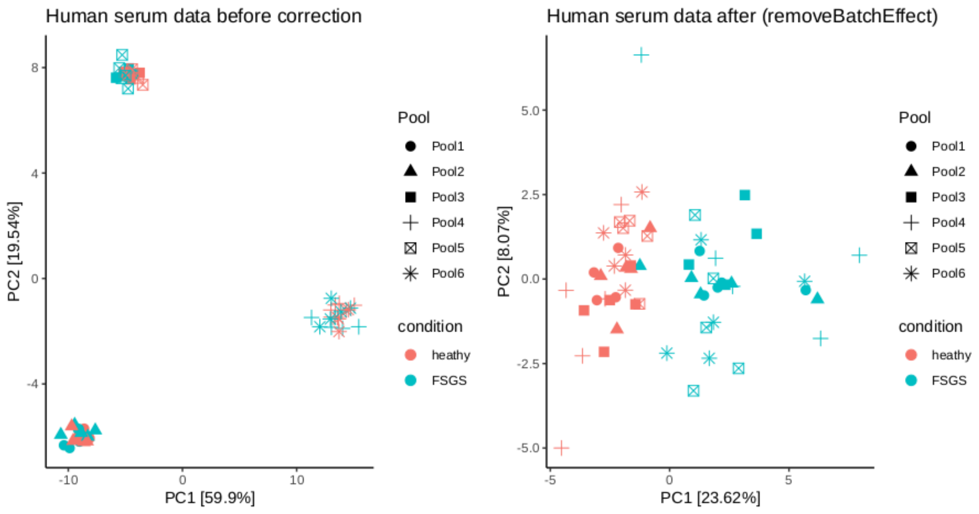

Supplementary Figure 11. Principal component analysis plots for all samples of bacterial (panel a) and human serum datasets (panel b) before and after batch effects correction using removeBatchEffect from the limma R package[4].

The datasets were preprocessed as described in the Methods.

For the bacterial dataset, samples measured by labs A and B (on the bottom right and left) were obtained from cell pellets, the other three labs (labs C, D, and E) worked with cell lysates prepared at the lab C.

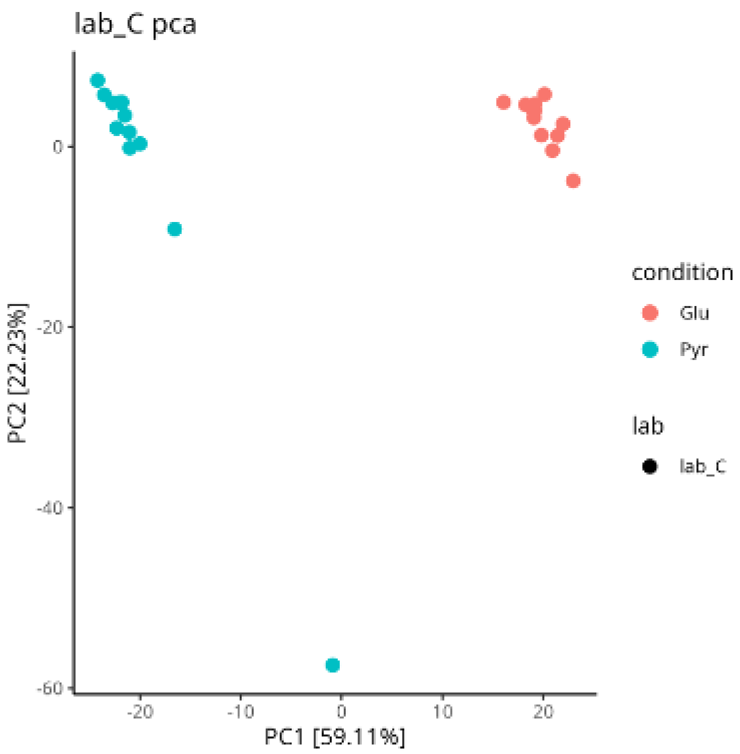

Supplementary Figure 12. Principal component analysis (PCA) plot for data from lab C with sample excluded after quality control step.

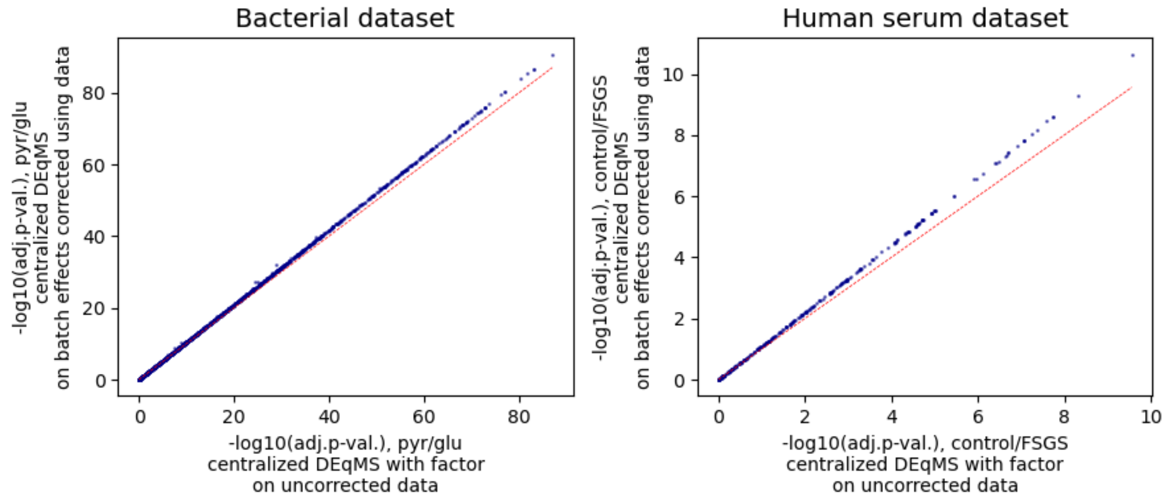

Supplementary Figure 13. The correlation for negative log-transformed adjusted p-values between results on aggregated data.

Plots with batch effect correction (y-axis) and without batch effect correction, but with batch effects included in the model (x-axis).

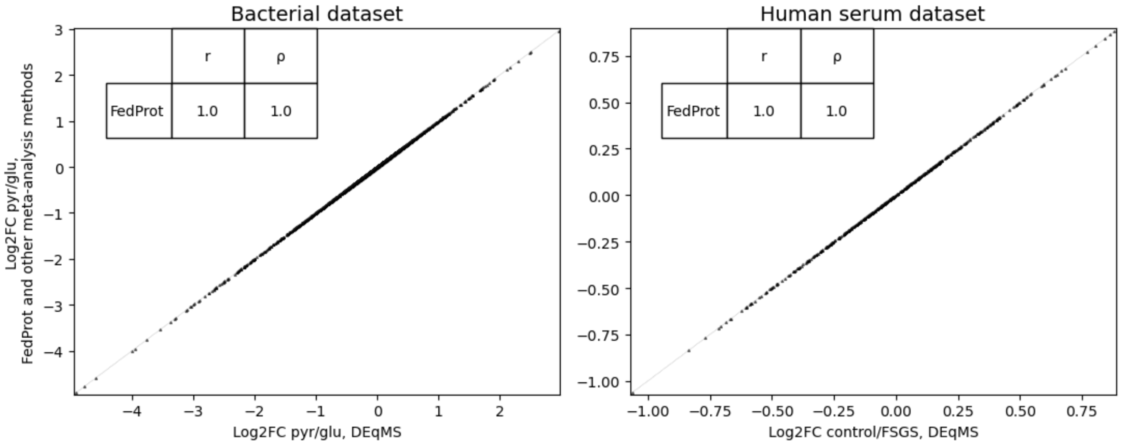

Supplementary Figure 14. The correlation for log-fold changes between central results on data after batch effect correction using removeBatchEffect (y-axis) and decentralized FedProt analysis (x-axis).

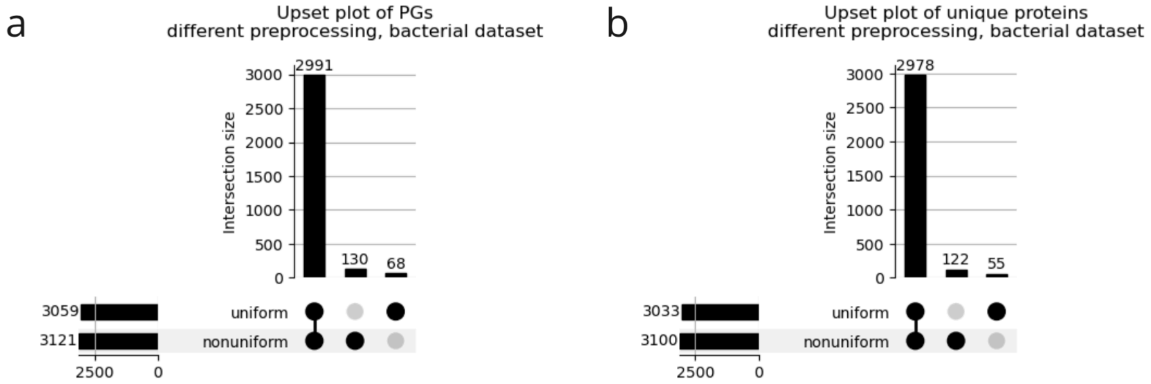

Supplementary Figure 15. **The number of unique PGs (panel a) and unique protein IDs (panel b) in quantification software outputs.**

Shown for uniform and non-uniform preprocessing for the bacterial dataset (preprocessed separately for 5 centers, union of all).

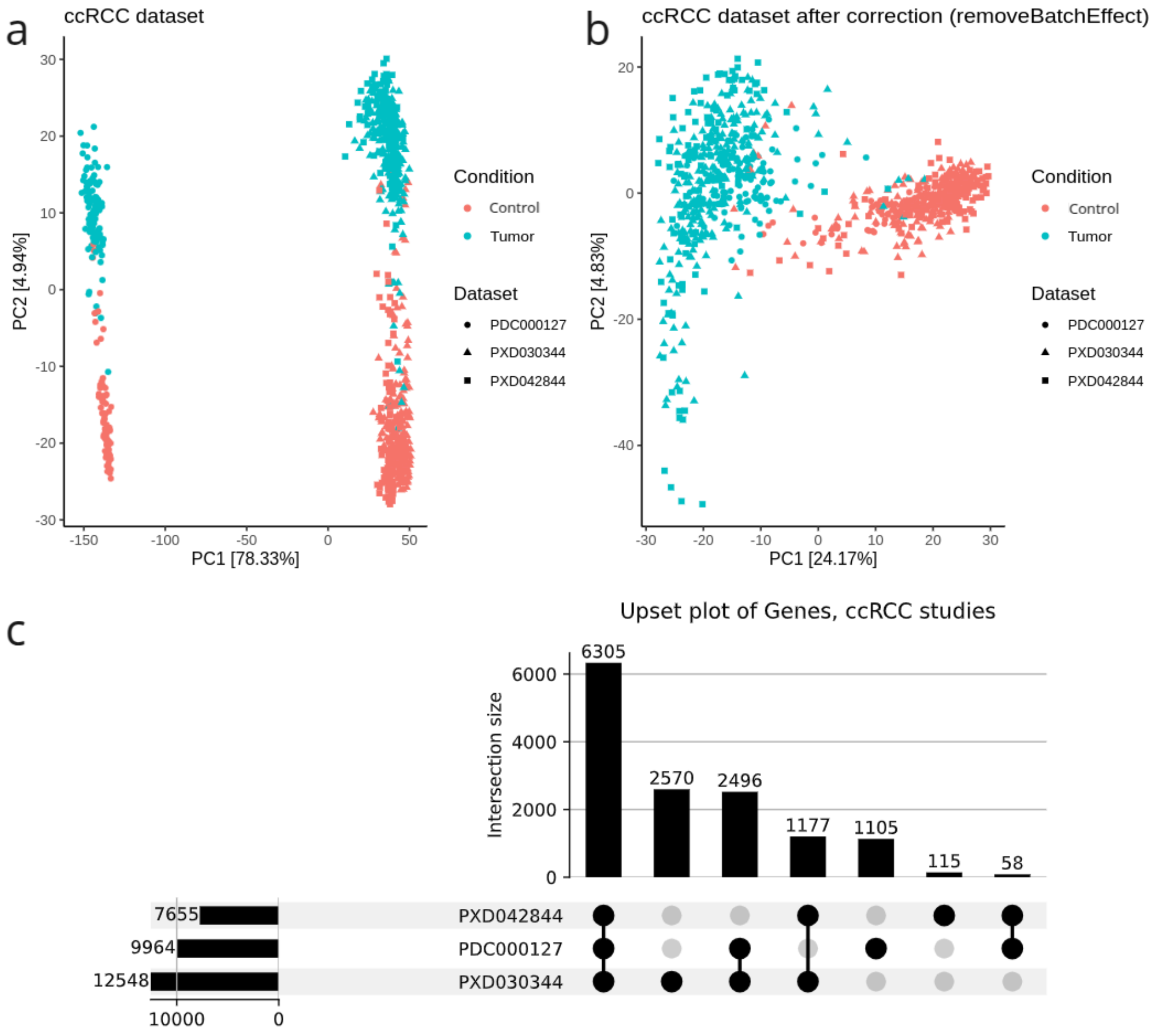

Supplementary Figure 16. Overview of the clear-cell renal cell carcinoma (ccRCC) dataset.

Panels a and b: principal component analysis (PCA) plots for data used for differential expression analysis (panel a) and after batch effect correction using limma removeBatchEffect function (panel b); panel c: the number of unique gene names in intensity matrices after filtering (keeping only rows that contain at least one value for each target class).

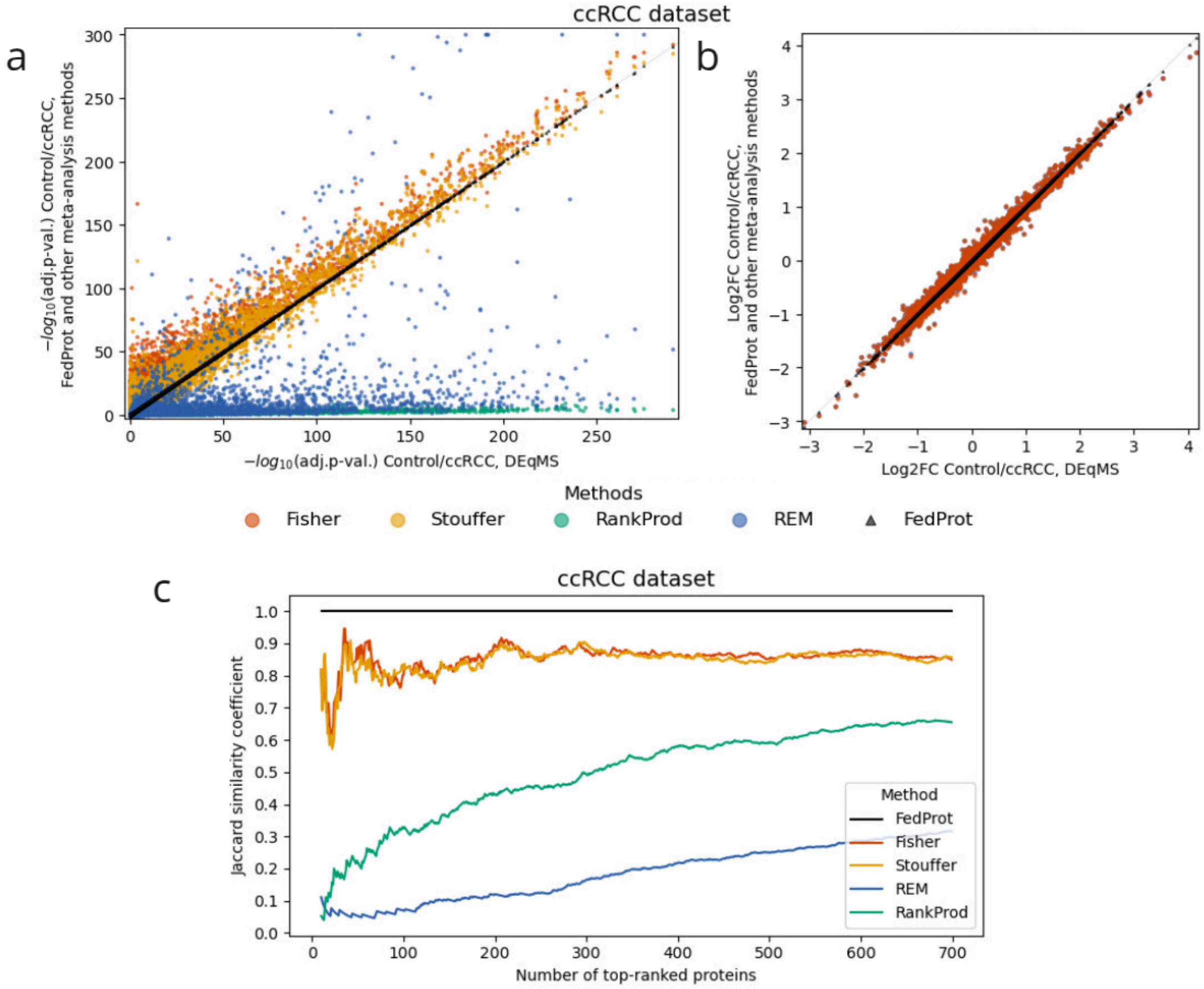

Supplementary Figure 17. **The performance of FedProt and meta-analyses compared to results of central DEqMS analysis with the ccRCC dataset (combination of 3 studies).**

Panels a and b: The comparison of negative log-transformed adjusted p-values (panel a) and logFC (panel b) computed by FedProt or meta-analysis methods with the centralized DEqMS analysis. The thin gray line is the diagonal.

Panel c: the dependency of the Jaccard similarity coefficient on the number of top-ranked proteins identified by the centralized DEqMS and decentralized approaches, proteins were ranked based on their decreasing negative log-transformed BH-adjusted p-values and not filtered by log2FC.

Metrics use each method's own |log2FC| and BH-adjusted p-values. The exact results (with p-values) are uploaded to the Zenodo[14].

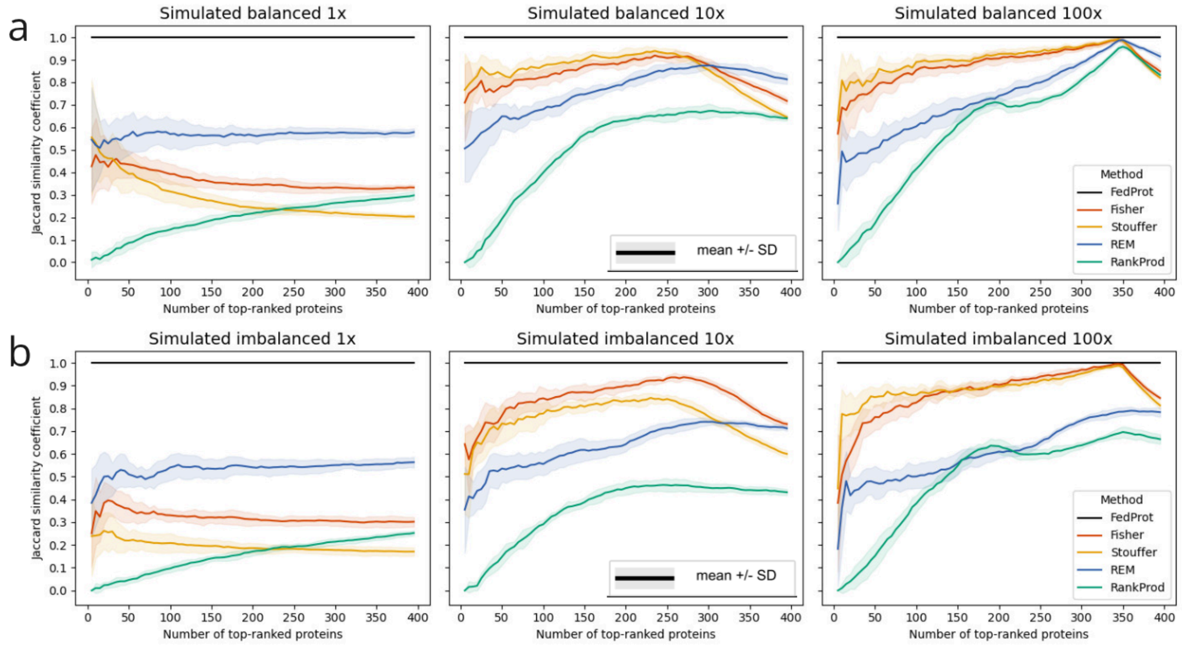

Supplementary Figure 18. Ranking consistency between centralized and decentralized methods for simulated datasets under different cohort sizes.
Panel a – simulated balanced datasets (1x, 10x, and 100x), panel b – simulated imbalance datasets (1x, 10x, and 100x). The dependency of the Jaccard similarity coefficient on the number of top-ranked proteins identified by the centralized *DEqMS* and decentralized approaches. Proteins were ranked based on their decreasing negative log-transformed BH-adjusted p-values and not filtered by log2FC. The generation of simulated data and the subsequent data analysis were repeated 10 times — aggregated results reported.
Metrics use each method's own |log2FC| and BH-adjusted p-values.

Supplementary Tables

Supplementary Table 1. The mean and maximum absolute differences between the log-fold change results of centralized *DEqMS* and FedProt or selected meta-analysis approaches.
The lowest differences are shown in bold font. The results for the Fisher's, Stouffer's, and the RankProd methods are the same because they use the same approach to estimate log-fold-changes.

Dataset	Method	Mean difference	Maximal difference
Bacterial	**FedProt**	**1.07E-14**	**5.72E-14**
	Fisher	0.003	0.191
	REM	0.017	0.233
Human serum	**FedProt**	**9.07E-15**	**3.54E-14**
	Fisher	0.010	0.146
	REM	0.019	0.133

Supplementary Table 2. Characteristics of the simulated datasets used to evaluate the effect of data imbalance.
Number of samples in each cohort (C1-C3) in each condition (A, B), for the confounder column — proportion of samples among condition B samples.

	**Cohorts**			**Condition A**			**Condition B**			**in B — frequency of samples with the confounder**		
	**C1**	**C2**	**C3**	**C1**	**C2**	**C3**	**C1**	**C2**	**C3**	**C1**	**C2**	**C3**
**Balanced**	200	200	200	100	100	100	100	100	100	0.6	0.6	0.6
**Mild imbalanced**	90	140	370	36	91	185	54	49	185	0.4	0.5	0.66
**Strong imbalanced**	40	80	480	32	28	288	8	52	192	0.2	0.5	0.7

Supplementary Table 3. The mean and maximum absolute differences between log-fold changes of centralized *DEqMS* and FedProt or selected meta-analysis approaches.

The lowest differences are shown in bold font. The generation of simulated data and the subsequent data analysis were repeated 50 times — mean and standard deviation for the mean absolute differences for these analyses results are provided.

Dataset	Method	Mean difference	Maximal difference
Simulated, balanced	FedProt	**6.62E-16 ± 8.60E-18**	**8.04E-15 ± 1.70E-15**
	Fisher	0.01 ± 1.51E-04	0.29 ± 0.23
	REM	0.03 ± 6.43E-04	1.85 ± 1.25
Simulated, mild imbalance	FedProt	**1.18E-15 ± 1.88E-17**	**1.42E-14 ± 2.25E-15**
	Fisher	0.09 ± 0.001	1.74 ± 0.83
	REM	0.04 ± 0.001	1.44 ± 0.57
Simulated, strong imbalance	FedProt	**2.50E-15 ± 3.86E-17**	**3.26E-14 ± 6.02E-15**
	Fisher	0.22 ± 0.003	5.27 ± 5.55
	REM	0.06 ± 0.002	2.41 ± 0.91

Supplementary Table 4. Quantification software used for preprocessing the bacterial dataset with an LC-MS/MS measurement overview.

Number of samples in each cohort in each condition. M9 is the medium used to grow *E. coli*. Quantifications were run in different research centers on different machines.

	Groups		Set-up	Quantification software
	M9 Pyruvate	M9 Glucose		
Lab A	10	10	Evosep One – Exploris 480	Spectronaut 17.5
Lab B	10	9	nanoElute – timsTOF Pro	DIA-NN 1.8
Lab C	9	10	Ultimate3000 – Orbitrap Fusion Lumos	DIA-NN 1.8
Lab D	10	10	EASY-nLC 1200 – Exploris 480	DIA-NN 1.8.1
Lab E	10	10	Ultimate3000 – QE-HFX	Spectronaut 17.2

Supplementary Table 5. Clear-cell renal cell carcinoma dataset description.

	Groups		Set-up
	Control	Tumor	
PDC000127	84	110	Orbitrap Fusion Lumos
PXD042844	114	115	Q Exactive HF-X
PXD030344	232	232	Q Exactive HF-X

Supplementary Table 6. DIA-NN v1.8.1 run parameters for the bacterial dataset.

Output will be filtered at 0.01 FDR
Precursor/protein x samples expression level matrices will be saved along with the main report
A spectral library will be generated
Deep learning will be used to generate a new in silico spectral library from peptides provided
Library-free search enabled
Min fragment m/z set to 200
Max fragment m/z set to 1800
N-terminal methionine excision enabled
In silico digest will involve cuts at K*,R*
Maximum number of missed cleavages set to 2
Min peptide length set to 7
Max peptide length set to 30
Min precursor m/z set to 360
Max precursor m/z set to 1800
Min precursor charge set to 1
Max precursor charge set to 4
Cysteine carbamidomethylation enabled as a fixed modification
Maximum number of variable modifications set to 1
Modification UniMod:35 with mass delta 15.9949 at M will be considered as variable
Modification UniMod:1 with mass delta 42.0106 at *n will be considered as variable
A spectral library will be created from the DIA runs and used to reanalyse them; .quant files will only be saved to disk during the first step
When generating a spectral library, in silico predicted spectra will be retained if deemed more reliable than experimental ones
DIA-NN will optimise the mass accuracy automatically using the first run in the experiment. This is useful primarily for quick initial analyses, when it is not yet known which mass accuracy setting works best for a particular acquisition scheme.
The following variable modifications will be scored: UniMod:1

Supplementary Table 8. Performance metrics calculated based on the results of FedProt and meta-analysis approaches for the bacterial dataset with non-uniform compared to centralized DEqMS results for the bacterial dataset with uniform and non-uniform preprocessing.

Jaccard similarity coefficients, and error rates (false positives and false negatives), for FP and FN, threshold of |logFC| > 0.5 and adj.p-value < 0.05 were used. The best performance is highlighted in bold.

Method	compared to uniform, centralized DEqMS			compared to non-uniform, centralized DEqMS		
	FP	FN	Jaccard similarity coefficient	FP	FN	Jaccard similarity coefficient
**FedProt**	**94**	**10**	**0.857**	**0**	**0**	**1**
Fisher	95	10	0.855	3	2	0.993
Stouffer	95	10	0.855	3	2	0.993
REM	95	13	0.851	14	16	0.959
RankProd	22	124	0.776	0	186	0.74

Supplementary Table 9. Performance metrics calculated based on the results of FedProt and selected meta-analysis approaches compared to centralized DEqMS results using the ccRCC dataset (combination of 3 studies).

Reported mean and maximum absolute differences in |log2FC|, negative log-transformed adjusted p-values, Jaccard similarity coefficients, and error rates (false positives and false negatives). For Jaccard similarity coefficients, FP and FN threshold of |logFC| > 0.5 and adj.p-value < 0.05 were used. The best performance is highlighted in bold.

Method	|LogFC|		Adj. p-values		FP	FN	Jaccard similarity coefficient
	Mean difference	Maximal difference	Mean difference	Maximal difference			
**FedProt**	**1.08E-15**	**8.44E-15**	**6.03E-14**	**7.11E-13**	**0**	**0**	**1**
**Fisher**	6.08E-02	6.35E-01	9.72E+00	1.63E+02	137	153	0.86
**Stouffer**	6.08E-02	6.35E-01	7.18E+00	1.18E+02	137	153	0.86
**REM**	6.11E-02	5.98E-01	2.52E+01	2.57E+02	51	391	0.78
**RankProd**	6.08E-02	6.35E-01	2.89E+01	2.86E+02	13	462	0.75

Supplementary Table 10. Characteristics of the simulated datasets used to evaluate the performance scaling with the number of samples.

Number of samples in each cohort (C1-C3) in each condition (A, B), for the confounder column — proportion of samples among condition B samples.

Dataset	Total samples	Cohorts			Condition A			Condition B			in B — frequency of samples with the confounder		
		C1	C2	C3	C1	C2	C3	C1	C2	C3	C1	C2	C3
Balanced													
1x	66	22	22	22	11	11	11	11	11	11	0.6	0.6	0.6
10x	660	220	220	220	110	110	110	110	110	110			
100x	6600	2200	2200	2200	1100	1100	1100	1100	1100	1100			
Strong imbalanced													
1x	66	12	8	46	9	3	31	3	5	15	0.2	0.5	0.7
10x	660	45	94	521	37	31	304	8	63	217			
100x	6600	450	940	5210	370	310	3040	80	630	2170			

Supplementary Table 11. Performance metrics calculated based on the results of FedProt and selected meta-analysis approaches compared to centralized DEqMS results using simulated datasets.

Mean and maximum absolute differences in negative log-transformed adjusted p-values, Jaccard similarity coefficients, and error rates (false positives and false negatives). Jaccard similarity coefficients and error rates were computed with $|\log2FC|$ > 1 and adj.p-value < 0.05 thresholds. The values corresponding to the best performance between all methods are highlighted in bold font. The generation of simulated data and the subsequent data analysis were repeated 10 times — the mean and standard deviation are reported.

Dataset	Method	Mean difference	Maximal difference	FP	FN	Jaccard similarity coefficient
**Balanced**						
Balanced 1x	FedProt	**8.93E-16 ± 1.19E-16**	**1.44E-14 ± 5.58E-15**	**0.0 ± 0.0**	**0.0 ± 0.0**	**1.00 ± 0.0**
	Fisher	0.05 ± 0.00	1.52 ± 0.38	0.20 ± 0.42	6.90 ± 4.48	0.17 ± 0.21
	Stouffer	0.06 ± 0.01	1.57 ± 0.33	0.0 ± 0.0	7.10 ± 4.46	0.16 ± 0.18
	REM	0.04 ± 0.00	1.82 ± 0.54	3.30 ± 1.64	1.80 ± 1.48	0.55 ± 0.17
	RankProd	0.67 ± 0.01	6.68 ± 1.29	237.00 ± 17.20	0.0 ± 0.0	0.04 ± 0.02
Balanced 10x	FedProt	**3.49E-15 ± 2.31E-16**	**2.08E-13 ± 7.19E-14**	**0.0 ± 0.0**	**0.0 ± 0.0**	**1.00 ± 0.0**
	Fisher	0.13 ± 0.01	13.80 ± 4.17	1.30 ± 0.95	1.50 ± 1.08	0.94 ± 0.03
	Stouffer	0.14 ± 0.01	10.30 ± 3.02	1.30 ± 0.95	1.70 ± 1.16	0.93 ± 0.03
	REM	0.16 ± 0.01	20.50 ± 3.37	3.70 ± 1.95	5.00 ± 1.89	0.81 ± 0.05
	RankProd	0.81 ± 0.02	33.20 ± 5.88	5.00 ± 2.21	0.50 ± 0.53	0.88 ± 0.04
Balanced 100x	FedProt	**1.77E-14 ± 8.88E-16**	**1.36E-12 ± 2.75E-13**	**0.0 ± 0.0**	**0.0 ± 0.0**	**1.00 ± 0.0**
	Fisher	0.55 ± 0.06	153.00 ± 38.30	1.80 ± 1.69	0.50 ± 0.85	0.92 ± 0.07
	Stouffer	0.47 ± 0.04	116.00 ± 27.90	1.80 ± 1.69	0.50 ± 0.85	0.92 ± 0.07
	REM	1.39 ± 0.13	206.00 ± 26.10	3.20 ± 2.15	1.90 ± 1.37	0.82 ± 0.06
	RankProd	4.92 ± 0.19	294.00 ± 4.44	1.90 ± 1.79	0.50 ± 0.85	0.92 ± 0.07
**Imbalanced**						
Imbalanced 1x	FedProt	**1.61E-15 ± 2.08E-16**	**2.26E-14 ± 5.88E-15**	**0.0 ± 0.0**	**0.0 ± 0.0**	**1.00 ± 0.0**
	Fisher	0.05 ± 0.00	1.56 ± 0.32	0.0 ± 0.0	9.30 ± 4.55	0.04 ± 0.05
	Stouffer	0.06 ± 0.00	2.04 ± 0.52	0.0 ± 0.0	9.70 ± 4.81	0.01 ± 0.02
	REM	0.05 ± 0.00	2.38 ± 0.77	2.60 ± 1.58	4.80 ± 4.64	0.41 ± 0.18
	RankProd	0.66 ± 0.01	6.86 ± 1.21	269.00 ± 9.96	0.70 ± 0.68	0.03 ± 0.02
Imbalanced 10x	FedProt	**1.48E-14 ± 1.78E-15**	**5.46E-13 ± 1.07E-13**	**0.0 ± 0.0**	**0.0 ± 0.0**	**1.00 ± 0.0**
	Fisher	0.15 ± 0.01	14.40 ± 4.70	20.50 ± 4.28	16.20 ± 4.24	0.43 ± 0.04
	Stouffer	0.32 ± 0.01	17.90 ± 2.47	18.60 ± 4.06	17.00 ± 4.67	0.43 ± 0.05
	REM	0.24 ± 0.01	30.50 ± 4.47	3.20 ± 1.23	7.50 ± 3.24	0.78 ± 0.06
	RankProd	0.95 ± 0.03	38.20 ± 3.93	71.10 ± 13.50	15.90 ± 4.41	0.25 ± 0.02
Imbalanced 100x	FedProt	**7.90E-14 ± 5.69E-15**	**5.99E-12 ± 7.93E-13**	**0.0 ± 0.0**	**0.0 ± 0.0**	**1.00 ± 0.0**
	Fisher	0.69 ± 0.08	231.00 ± 42.10	16.50 ± 3.63	9.40 ± 3.60	0.40 ± 0.08
	Stouffer	1.86 ± 0.06	112.00 ± 14.40	16.50 ± 3.63	9.40 ± 3.60	0.40 ± 0.08
	REM	2.62 ± 0.09	252.00 ± 17.90	5.10 ± 2.28	4.30 ± 1.83	0.70 ± 0.06
	RankProd	5.80 ± 0.15	296.00 ± 0.17	17.20 ± 3.29	9.40 ± 3.60	0.40 ± 0.08

Supplementary Table 12. Metadata (clinical characteristics) for the human serun dataset with populational characteristics.

Patient ID	Included in which research center?	Group	Sex	Age	Proteinuria [mg/g creatinine]	eGFR [ml/min]
FSGS1	Center 1	FSGS	f	45	6000	97
FSGS2	Center 1	FSGS	m	31	16000	130
FSGS3	Center 1	FSGS	m	35		41
FSGS4	Center 1	FSGS	m	73	4510	38
FSGS5	Center 1	FSGS	m	70	15616	56
FSGS6	Center 1	FSGS	m	35	1000	88
FSGS7	Center 1	FSGS	m	51	6850	36
FSGS8	Center 1	FSGS	f	56	1904	60
FSGS9	Center 1	FSGS	f	20	13580	136
FSGS10	Center 1	FSGS	m	74	10282	24
Ctrl1	Center 1	Ctrl	f	35		
Ctrl2	Center 1	Ctrl	m	65		
Ctrl3	Center 1	Ctrl	m	32		
Ctrl4	Center 1	Ctrl	f	64		
Ctrl5	Center 1	Ctrl	m	56		
Ctrl6	Center 1	Ctrl	m	24		
Ctrl7	Center 1	Ctrl	f	23		
Ctrl8	Center 1	Ctrl	m	36		
Ctrl9	Center 1	Ctrl	f	42		
Ctrl10	Center 1	Ctrl	m	33		
FSGS11	Center 2	FSGS	m	48	8000	53
FSGS12	Center 2	FSGS	f	30	2100	137
FSGS13	Center 2	FSGS	m	41	10000	32
FSGS14	Center 2	FSGS	f	29	11476	34
FSGS15	Center 2	FSGS	m	60	8028	12
FSGS16	Center 2	FSGS	f	55	8094	55
FSGS17	Center 2	FSGS	m	51	20000	68
FSGS18	Center 2	FSGS	f	76	22565	21
FSGS19	Center 2	FSGS	f	23	3276	75
FSGS20	Center 2	FSGS	f	29	4000	125
Ctrl11	Center 2	Ctrl	f	28		

Patient ID	Included in which research center?	Group	Sex	Age	Proteinuria [mg/g creatinine]	eGFR [ml/min]
Ctrl12	Center 2	Ctrl	f	33		
Ctrl13	Center 2	Ctrl	f	33		
Ctrl14	Center 2	Ctrl	m	26		
Ctrl15	Center 2	Ctrl	m	25		
Ctrl16	Center 2	Ctrl	f	66		
Ctrl17	Center 2	Ctrl	f	50		
Ctrl18	Center 2	Ctrl	f	42		
Ctrl19	Center 2	Ctrl	f	26		
Ctrl20	Center 2	Ctrl	f	24		
FSGS21	Center 3	FSGS	f	29	4000	125
FSGS22	Center 3	FSGS	m	55	5000	35
FSGS23	Center 3	FSGS	m	55	20000	10
FSGS24	Center 3	FSGS	m	34	6000	108
FSGS25	Center 3	FSGS	m	35	3300	58
FSGS26	Center 3	FSGS	m	66	8000	0
FSGS27	Center 3	FSGS	f	44	796	113
FSGS28	Center 3	FSGS	f	37	5801	64
FSGS29	Center 3	FSGS	m	70	7248	29
FSGS30	Center 3	FSGS	m	37	3847	104
Ctrl21	Center 3	Ctrl	m	79	12152	15
Ctrl22	Center 3	Ctrl	m	35	58	103
Ctrl23	Center 3	Ctrl	f	49	55	70
Ctrl24	Center 3	Ctrl	f	61	0	98
Ctrl25	Center 3	Ctrl	f	52	73	101
Ctrl26	Center 3	Ctrl	m	60	58	92
Ctrl27	Center 3	Ctrl	m	53	67	89
Ctrl28	Center 3	Ctrl	f	65	149	78
Ctrl29	Center 3	Ctrl	m	65	52	50
Ctrl30	Center 3	Ctrl	f	23	0	125

Supplementary Table 13. **Variable window scheme for data-independent acquisition in Lab C**.
z = charge state, m/z = window center, Isolation window (m/z) = window width.

centered m/z	z	Isolation Window width (m/z)
375	2	30
399	2	20
413.5	2	11
423.5	2	11
433.5	2	11
443.5	2	11
453.5	2	11
463.5	2	11
473.5	2	11
483.5	2	11
493.5	2	11
503.5	2	11
513.5	2	11
523.5	2	11
533.5	2	11
543.5	2	11
553.5	2	11
563.5	2	11
573.5	2	11
583.5	2	11
593.5	2	11
603.5	2	11
613.5	2	11
623.5	2	11
635.5	2	15
649.5	2	15
663.5	2	15
677.5	2	15
691.5	2	15
705.5	2	15
722	2	20
741	2	20
760	2	20
781.5	2	25

centered m/z	z	Isolation Window width (m/z)
805.5	2	25
834.5	2	35
871	2	40
920	2	60
991.5	2	85
1166.5	2	267

Supplementary Methods

Meta-analysis approaches

In order to evaluate FedProt's accuracy in comparison to meta-analyses, we used three classes of meta-analyses: effect size combination methods, p-value combination methods (Fisher's method[15] and Stouffer's method[16]), and non-parametric rank combination methods[17,18].

As methods based on integration of p-values we used Fisher's method[15] and Stouffer's method[16]. In these methods, p-values obtained from each individual analysis can be integrated into a single combined p-value per protein or gene assuming the sum, minimum or maximum of log-transformed p-values from independent studies follow a certain distribution[17].

Fisher's method is a classical method[15,19] in which the meta-p-values are calculated based on a chi-squared distribution. It is a common method for omics data analysis, but it is sensitive to very small p-values[17] and treats large and small p-values asymmetrically[20]. We used the Fisher's method implementation available in the *metaVolcanoR* package[21].

On the other hand, Stouffer's method[16,19], also known as normal, Z-method, or Z-transform test, integrates p-values but allows for different study weights and has more power and more precision than Fisher's method[20]. We used the Stouffer's method implementation from the *MetaDE* package[22].

For effect size combination methods, we used the Random Effects Model (REM)[17,18] implementation from *metaVolcanoR* package[21]. REM takes into account the heterogeneity between studies by adding a between-study variance[23]. But estimating this variance can be challenging, especially with a small number of studies (cohorts). This is because REM computes p-values using global effect sizes, assuming a normal distribution.

Furthermore, we used the Rank Product method as a representative of non-parametric rank combination methods. RankProd from the *RankProd* R package is a non-parametric rank-based approach, the significance is assessed by a nonparametric permutation test[24]. While it is non-parametric and doesn't require homogeneity of variances, it might be less powerful than parametric methods when their assumptions are met.

For all chosen meta-analysis methods except REM, global fold-change was calculated as the mean of local fold-changes, producing the same values. Consequently, only Fisher's method and REM results were utilized for the evaluation of log-fold changes.

Human serum dataset – LC-MS/MS measurement

Sample preparation

The samples were separated into three groups, each containing 10 healthy and 10 FSGS samples, blinded and distributed to three studies centers by the clinical partners. Sample's metadata is provided in **Supplementary Table 12.**

Sample preparation for mass spectrometry

Samples were prepared by three independent scientists applying a harmonized protocol. Briefly, 10 µL of the serum samples were loaded onto depletion columns (High-Select Top14 Abundant Protein Depletion Resin, Thermo Fisher Scientific) to deplete the 14 most abundant serum proteins. Thirty micrograms of the filtrates were reduced, alkylated, and digested using LysC followed by trypsin, applying the filter-aided sample preparation (FASP) protocol by Wisniewski[25]. The samples were subsequently desalted using Oasis HLB 96-well µElution Plates (Waters) and reconstituted in 30 µL of 0.1% formic acid containing 3% acetonitrile. The resulting peptide concentrations were determined using a NanoDrop Microvolume Spectrophotometer (Thermo Fisher Scientific). Subsequently, five micrograms of each sample, along with a pooled common reference sample, were labeled using the TMT-11plex kit (Thermo Fisher Scientific). TMT-labeled samples were combined into six sets, each containing five healthy, five FSGS, and one common reference sample, and dried in vacuum. Ultimately, the six sample sets were fractionated (High pH reversed phase peptide fractionation kit, Thermo Fisher Scientific), and the fractions were dried in vacuum.

Mass spectrometry data were acquired in three independent research centers using their preferred instruments and corresponding parameter setups (**Main text Table 1B**).

LC-MS/MS measurement

Mass spectrometry location 1 — QExactive HF

Prior to measurement, all samples were dissolved in 0.1 % formic acid and injected into an Easy-nLC 1200 coupled to a Q Exactive HF mass spectrometer (both Thermo Fisher Scientific). Samples were loaded onto a 20-cm analytical HPLC column (75 µm ID Pico Tip fused silica emitter, New Objective) packed in-house using ReproSil-Pur C18-AQ 1.9-µm silica beads (Dr. Maisch GmbH) and separated in a 120-min multistep gradient ranging from 10% solvent B to 90% solvent B (0.1 % formic acid in acetonitrile) at a constant flow rate of 200 nL/min. The nano-HPLC column was drawn to a tip of ~10 µm and acted as the electrospray needle of the MS source. Samples were measured in data-dependent mode, applying a MS/MS scan to the Top 10 most abundant precursors per survey scan and a dynamic exclusion of 30 s. HCD collision energy was set to 34% with an isolation width of 0.7 Da. Survey

scans were acquired in a scan range of 300–1650 m/z, a mass resolution of 60,000, an AGC target value of 3 × $10^{6,}$ and a maximum injection time of 50 ms. For MS/MS scans, AGC target and maximum injection time were set to 1 × 10^{5} and 110 ms, respectively.

Mass spectrometry location 2 — Exploris480

Samples were dissolved in 0.1% formic acid and analyzed by online C18 nanoHPLC-MS/MS with a system consisting of an Ultimate3000 nano gradient HPLC system (Thermo, Bremen, Germany), and an Exploris480 mass spectrometer (Thermo Fisher Scientific). Fractions were injected onto a cartridge precolumn (300 μm × 5 mm, C18 PepMap, 5 um, 100 A, and eluted via a homemade analytical nano-HPLC column (30 cm × 75 μm; Reprosil-Pur C18-AQ 1.9 μm, 120 A (Dr. Maisch, Ammerbuch, Germany). The gradient was run from 2% to 36% solvent B (20/80/0.1 water/acetonitrile/formic acid (FA) v/v) in 120 min at 250 nl/min. The nano-HPLC column was drawn to a tip of ~10 μm and acted as the electrospray needle of the MS source. The mass spectrometer was operated in data-dependent MS/MS mode with a cycle time of 3 s, with a HCD collision energy at 36% and recording of the MS2 spectrum in the Orbitrap, with a quadrupole isolation width of 1.2 Da. In the master scan (MS1), the resolution was 120,000, the scan range 350-1600, at an AGC target of standard maximum fill time of 50 ms. A lock mass correction on the background ion m/z=445.12003 was used. Precursors were dynamically excluded after n=1 with an exclusion duration of 45 s, and with a precursor range of 30 ppm. Charge states 2-5 were included. For MS2, the first mass was set to 110 Da, and the MS2 scan resolution was 45,000 at an AGC target of 200% fill time of 'auto'.

Mass spectrometry location 3 — Fusion LUMOS

TMT-labeled peptides were dissolved in 0.1% formic acid and subsequently analyzed by on-line C18 nanoHPLC-MS/MS with a system consisting of an Easy nLC 1200 gradient HPLC system (Thermo, Bremen, Germany) and an Orbitrap Fusion LUMOS mass spectrometer (Thermo). Fractions were injected onto a homemade precolumn (100 μm × 15 mm; Reprosil-Pur C18-AQ 3 μm, Dr. Maisch, Ammerbuch, Germany) and eluted via a homemade analytical nano-HPLC column (30 cm × 75 μm; Reprosil-Pur C18-AQ 1.9 μm). The analytical column temperature was maintained at 50 °C with a Sonation PRSO-V2 column oven. The gradient was run from 2% to 36% solvent B (20%/80%/0.1% water/acetonitrile/formic acid (FA) v/v) in 120 min. The nano-HPLC column was drawn to a tip of ~10 μm and acted as the electrospray needle of the MS source. The mass spectrometer was operated in data-dependent MS/MS mode with a cycle time of 3 s, with a HCD collision energy at 36% and recording of the MS2 spectrum in the Orbitrap, with a quadrupole isolation width of 1.2 Da. In the master scan (MS1), the resolution was 120,000, the scan range 350-1600, at an AGC target of 'standard' maximum fill time of 50 ms. A lock mass correction on the background ion m/z=445.12003 was used. Precursors were dynamically excluded after n=1 with an exclusion duration of 45 s, and with a

precursor range of 20 ppm. Charge states 2-5 were included. For MS2, the first mass was set to 110 Da, and the MS2 scan resolution was 50,000 at an AGC target of 200% fill time of 50 ms.

Bacterial dataset – LC-MS/MS measurement

Sample preparation

We evaluated FedProt using an LFQ dataset of 118 samples generated from *Escherichia coli* MG1655 (DSM 18039) cultures. Single colonies from passage three were inoculated in 5 ml either M9 Pyruvate (1x M9 salts, 2 mM $MgSO_4$, 0.1 mM $CaCl_2$, 40 mM sodium pyruvate) or M9 Glucose medium (1x M9 salts, 2 mM $MgSO_4$, 0.1 mM $CaCl_2$, 20 mM glucose) and grown at 37 °C overnight. Of these overnight cultures, 100 µl culture was inoculated in 10 mL fresh M9 pyruvate or M9 glucose medium. Cells were incubated at 37 °C with shaking at 200 rpm and harvested after six hours (M9 glucose) or 12 hours (M9 pyruvate). For cell pellets, cells were centrifuged and medium was removed (no further washing step executed). For cell lysates, after removal of the medium, cells were lysed in 50 µl 100% TFA for 5 minutes at 55 °C, and the solution neutralized with 450 µl 2 M Tris. Samples were shipped on dry ice either as lysates or as cell pellets (**Main text Table 1A**).

Lab A and Lab B received cell pellets, while others (C, D, E) received already lysed cells.

Sample preparation for mass spectrometry

Bacterial cell lysis.

Lab A. 50µL LYSE buffer (from iST kit, PreOmics, Martinsried) were added to the bacterial cell pellets, and samples were incubated for 10 minutes at 95°C with shaking at 1000 rpm. For lysis, samples were sonicated in a Bioruptor Pico for 10 cycles of 30 seconds on/30 seconds off at 4°C.

Lab B. Bacterial cells were lysed according to the SPEED protocol[26] with further adaptation[27]. As mentioned above, 50 µl 100% TFA were added to every sample with subsequent incubation at 55°C for 5 minutes. Subsequently, 450 µl 2M Tris was added to the cell lysates to neutralize the sample.

Labs C-E. The laboratories C, D, and E used cell lysates prepared following the same protocol as lab B, except that the lysis was performed by lab C and the cell lysates were sent to labs D and E and diluted.

Protein digestion and peptide purification.

Each lab used slightly different protocols for protein digestion, peptide purification, and preparation for MS.

Lab A. Sample preparation with the iST kit. Protein concentrations of lysates were determined using bicinchoninic acid (BCA) assay (Pierce, #23252). For sample preparation for MS, 50 µg protein per sample was filled up to 50µL with LYSE buffer. Samples were then incubated at 95°C for 10 minutes (shaking at 1000 rpm). Then the iST protocol was used for all samples according to the manufacturer's guidelines (PreOmics GmbH, Martinsried). Briefly, after reduction and alkylation (LYSE buffer), trypsin and Lys-C were added for digestion, and the samples were incubated for 3 hours at 37°C, shaking at 500 rpm. Digestion was stopped by adding the STOP

buffer. Resulting peptides were cleaned up on the CARTRIDGE and then eluted. The peptide solution was dried in a Concentrator Plus (Eppendorf), and the resulting peptide pellet resuspended in 100µL LC-LOAD buffer.

Solid phase extraction using Evotips. For prefractionation on the Evosep One system, 1 µL of resuspended peptides (approx. 0.5µg per Evotip) were loaded on Evotips. Briefly, Evotips were first rinsed with 20µL of solvent B (0.1% FA/ACN, centrifugation at 800g, 60 s) and the C18 material conditioned in isopropanol for 30 seconds. The C18 material was equilibrated with 20µL solvent A (0.1% FA/H2O) before the sample (diluted in solvent A) was loaded, and the tips were washed two times with solvent A.

Lab B. In-solution tryptic digestion. Protein concentrations of cell lysates were determined using bicinchoninic acid (BCA) assay (Interchim Uptima, Paris, France). 10 µg of protein were used for tryptic digestion. Proteins were reduced and alkylated by additions of 100mM Tris(2-carboxyethyl)phosphine (TCEP) and 440 mM chloroacetic acid (CAA). Samples, covered with aluminum foil, were then incubated at 95°C (5 minutes, shaking at 400 rpm). Next, samples were diluted from 2 M to 1M Tris and cooled. Finally, overnight proteolytic digestion at 37°C (shaking at 400 rpm) was performed by adding trypsin at a protease to protein ratio of 1:50.

Solid phase extraction using StageTips. Samples were acidified to pH < 3. In-house built C18 Stage Tips were equilibrated with 250 µl 100% ACN and washed with 250 ul elution solution (40% ACN, 0.1% FA), followed by 250 µl washing solution (0.1% FA). Next, the digests were loaded onto the column, and the stage tips were centrifuged (1-5 min at 2000 x g, making sure that none of the tips dried out) and washed with 250 µl washing solution (0.1% FA). Finally, peptides were eluted twice with 40 µl elution solution (40% ACN, 0.1% FA). Samples were dried in a SpeedVac at 35°C and reconstituted in 50 µl 0.1% FA.

Lab C. In-solution Tryptic Digestion. This step was performed similarly to Lab B, but with variations in TCEP (9 mM) and CAA (33 mM) concentrations, and a heated cap was used during incubation.

Solid Phase Extraction using StageTips[28]. Samples were acidified (pH < 3 with 6% formic acid). The in-house build C18 StageTips, with three Empore C18 (3M) disks, were equilibrated consecutively with 250 µl 100% ACN, 250 ul elution solution (40% ACN, 0.1% FA), and 250 µl washing solution (2% ACN, 0.1% FA; each step: 2 min at 211 x g). Next, the digested sample was loaded onto the StageTip, centrifuged (5 min at 211 x g), and afterwards washed (2% ACN, 0.1% FA; 2 min at 211 x g). Finally, peptides were eluted twice with 50 µl elution solution (40% ACN, 0.1% FA; 2 minutes at 500 x g). All samples were dried in a centrifugal evaporator (Centrivap Cold Trap -50, Labconco, US) and stored at -80°C.

Lab D. Protein Digestion. Proteins were reduced and alkylated using 4 µL of 100 mM TCEP and CAA, respectively, and incubated at 95°C, shaking at 400 rpm, for 5 minutes. Lysate dilution was to 1M Tris using ddH_2O. Trypsin digestion (0.2 µg, 1:50 ratio) was done by overnight incubation at 37°C with shaking at 400 rpm. The digest was quenched at a final concentration of 3% FA.

Solid phase extraction using StageTips. Peptides were purified using the in-StageTip protocol[28] and styrenedivinylbenzene reverse-phase sulfonate (SDB-RPS, Empore™ SPE Disks, CDS Analytical, 98-0604-0226-4). In brief, a total of 20 µg of peptides was loaded on the stage tips (500 x g, 10 minutes). Peptides were washed twice using 1% TFA (v/v) in isopropanol and once using 0.2% TFA in MS-grade H2O (1000 x g). Peptides were then eluted (80% acetonitrile (v/v), 1% NH4+ (v/v) in MS-grade H_2O) at 300 x g and dried (60 minutes, 45°C, SpeedVac centrifuge, Eppendorf). Samples were resuspended (0.1% (v/v) TFA, 2% acetonitrile in MS-grade H_2O) and stored at -20°C.

Lab E. In-solution Tryptic Digestion was performed using the same protocol as in Lab B.

Solid Phase Extraction Using SDBRPS StageTips[29]. Samples were acidified to a pH<3 with FA. The in-house built SDBRPS StageTips using two Empore SDBRPS (3M) disks were equilibrated consecutively with 100 µl 100% ACN, 100 µl 30% MeOH, 1% TFA and 150 µl washing solution 3 (0.2% TFA; each step: 1 min at 800 x g). Next, the digested sample was diluted in 200µl 1% TFA and loaded onto the StageTip (1 min, 800 x g). Then, StageTips were washed three times. With 100µl washing solution 1 (99% ethyl acetate and 1% TFA, 1 min, 800 x g); with 100µl washing solution 2 (99% isopropanol and 1% TFA, 1 min, 800 x g), with 150µl washing solution 3 (2 min at 800 x g). Finally, peptides were eluted with 60 µl elution solution (80% ACN, 5% from 25% NH_4OH, 2 min, 800 x g). Post drying, samples were stored at -20°C.

LC-MS/MS measurement

Mass spectrometry data were acquired in five independent research centers using their preferred instruments and corresponding parameter setups (Main text **Table 1A**).

Lab A. Samples were analyzed in a randomized injection manner on an Evosep One LC using the 30 spd (samples per day) method using a 15cm x 150µm x 1.5µm column from PepSep heated to 40°C in a column oven (Sonation GmbH). Eluted peptides were electrosprayed into an Exploris 480 mass spectrometer (Thermo Fisher Scientific, Bremen). The MS was operated in a data-independent acquisition mode. MS1 spectra (380-980 m/z) were recorded at a resolution of 120,000 using an automatic gain control (AGC) target value of 300% and a maximum injection time of 100 ms. MS2 spectra were acquired at a resolution of 30,000, with an automatic gain control (AGC) target value of 3000% and auto maximum injection time. Isolation windows were 20 m/z with an overlap of 1 m/z, resulting in 30 windows. Normalized collision energy was set to 30%, and data were acquired in centroid mode.

Lab B. Samples (3 µl, around 300 ng of peptides) were analyzed on a nanoElute LC coupled to a timsTOF Pro mass spectrometer with a CaptiveSpray ion source (Bruker, Germany). Samples were injected on a self-packed C18 column (75µm internal diameter) with 1.9 µm ReproSil-Pur 120 C18-AQ resin (Dr Maisch,

Germany). A gradient of water (A) and acetonitrile (B) supplemented with 0.1% formic acid was applied at a flow rate of 300 nL/min and a column temperature of 50°C. The following gradient was applied: 0 min, 2% B; 2 min, 5% B; 62 min, 24% B; 72 min, 35% B; 75 min, 60% B; 78 min, 85% B. The MS was operated in a data-independent acquisition parallel accumulation-serial fragmentation (PASEF) mode. Ion accumulation and separation using trapped ion mobility spectrometry (TIMS) was set to a ramp time of 100 ms. One scan cycle included one TIMS full MS scan and two rows of 30 windows with a width of 25 m/z covering a range of 400-1,150 m/z. 5 scans per PASEF scan were applied.

Lab C. Around 500 ng peptides dissolved in washing solution (2% ACN, 0.1% FA) were analyzed on a Dionex Ultimate 3000 RSLCnano system coupled to an Orbitrap Fusion Lumos Tribrid Mass Spectrometer (Thermofisher Scientific, Bremen). Injected peptides were delivered to a trap column (ReproSil-pur C18-AQ, 5 µm, Dr. Maisch, 20 mm × 75 µm, self-packed) at a flow rate of 5 µL/min in 100% solvent A (0.1% formic acid in HPLC grade water). After 10 min of loading, peptides were transferred to an analytical column (ReproSil Gold C18-AQ, 3 µm, Dr. Maisch GmbH, 400 mm × 75 µm, self-packed) and separated using a 60 min linear gradient from 4% to 34% of solvent B (0.1% formic acid in acetonitrile and 5% (v/v) DMSO) at 300 nL/min flow rate. Both nanoLC solvents contained 5% (v/v) DMSO. The Fusion Lumos Tribrid Mass Spectrometer was operated in data-independent acquisition and positive ionization mode. MS1 spectra (360–1300 m/z) were recorded at a resolution of 60,000 using an automatic gain control (AGC) target value of 1e6 and maximum injection time (maxIT) of 50 ms. MS2 spectra were acquired at a resolution of 30,000, a scan range of 200-1,800 m/z, and with an automatic gain control (AGC) target value of 5E5 and maximum injection time (maxIT) of 54 ms. We used a variable window acquisition scheme with 40 windows overlapping by 1 m/z (see **Supplementary Table 13**) with a default charge state of two. Fragmentation was performed using higher energy collision induced dissociation (HCD) and a normalized collision energy of 30%.

Lab D. MS data were acquired on an EASY-nLC 1200 ultrahigh-pressure system (Thermo Fisher Scientific, San Jose, USA) coupled to an Orbitrap Exploris 480 Mass Spectrometer (Thermo Fisher Scientific, Waltham, USA) using a nano-electrospray ion source (Thermo Fisher Scientific). A total of 200 ng peptides was injected into a 50 cm column (inner diameter: 75µm, generated in-house[29] using ReproSil-Pur C18-AQ 1.9µm beads from Dr. Maisch GmbH, Ammerbuch, Germany). The temperature was kept constant at 55°C in a column oven. A two-buffer system enabled the gradual elution of peptides: buffer A (0.1% FA in H_2O) and buffer B (80% acetonitrile, 0.1 % FA in H_2O). During the course of liquid chromatography (LC), buffer B was increased from 2% to 35% within the first 60 minutes, followed by a further increase to 50% within 6 min, to 60% within 4 min and to 90% within 1 min which was kept constant for 4 min to ensure a complete elution of peptides. The flow rate was kept constant at 300 nl/min. DIA of the MS experiments included MS1 scans (scan range: 300 to 1,650 m/z; resolution: 120,000; maximum injection time: 20 ms; normalized AGC target: 300%) as well as sequential

MS2 scans (resolution: 30,000; maximum injection time: 54 ms; normalized AGC (Automatic Gain Control) target: 3000%) using 44 DIA isolation windows. Peptides were fragmented using stepped HCD collision energies (25, 27.5, 30).

Lab E. The MS data were acquired in DIA mode on a QExactive-HFX mass spectrometer (Thermo Fisher Scientific Inc., Waltham, MA, USA). Around 400 ng per sample were automatically loaded to the online coupled RSLC (Ultimate 3000, Thermo Fisher Scientific Inc.) HPLC system. A Nano-Trap column was used (300-µm inner diameter (ID) × 5 mm, packed with Acclaim PepMap100 C18, 5µm, 100 Å from LC Packings, Sunnyvale, CA, USA), before separation by reversed-phase chromatography (Acquity UPLC M-Class HSS T3 Column 75µm ID × 250 mm, 1.8µm from Waters, Eschborn, Germany) at 40°C. Peptides were eluted from the column at 250 nl/min using increasing ACN concentration in 0.1% formic acid from 3 to 40% over a 95-min gradient. The DIA method consisted of a survey scan from 300 to 1,500 m/z at 120,000 resolution and an automatic gain control (AGC) target of 3×10^6 or 120-ms maximum injection time. Fragmentation was performed via higher-energy collisional dissociation with a target value of 3×10^6 ions determined with predictive AGC. Precursor peptides were isolated with 37 variable windows spanning from 300 to 1,650 m/z at 30,000 resolution with an AGC target of 3×10^6 and automatic injection time. The normalized collision energy was 28, and the spectra were recorded in profile type.

Data exchange using the FeatureCloud infrastructure

Supplementary Figure 19 (adapted from Matschinske et al., 2023[30]) provides an overview of the communication process in the collaborative analysis workflow within the FeatureCloud platform. The process can involve additive secret sharing or the direct transmission of parameters.

After a step is completed, each client has local parameters (X_i) that need to be sent to the coordinator. When using additive secret sharing (green arrows, **Supplementary Figure 19**), each client generates n randomly sampled masks, r_1, …, r_n, as equally distributed random values, and splits the data to be sent into n pieces. These masks are used to create noisy data representations (X_i - r_1 - … - r_n). The masked data and masks are encrypted using the public keys of other clients to prevent interception. The encrypted pieces are then exchanged among clients via a relay server, which acts as an intermediary, ensuring no single client receives more than one piece of data from another. After decrypting the received pieces using their private keys, each client sums the data and re-encrypts the result. This re-encrypted sum is then sent through the relay server to the coordinator, who decrypts and aggregates the sums to compute the global result.

Alternatively, when secure aggregation is unnecessary, such as transmitting non-sensitive information like protein group names, parameters are sent directly to the coordinator without additive secret sharing (green arrows, **Supplementary Figure 19**). After updating, the global data is sent back to the clients for the next steps (black arrows, **Supplementary Figure 19**). For further details, see Matschinske et al., 2023[30].

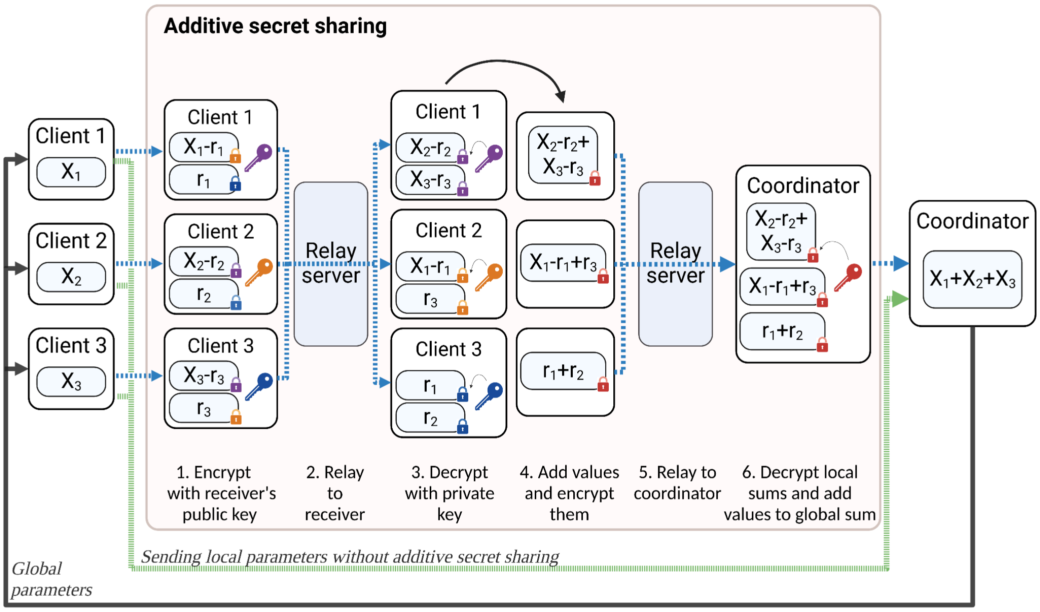

Supplementary Figure 19. An overview of the communication process during FeatureCloud app run (adapted from Matschinske et al., 2023[30]).
The figure is shown given n=1. In FedProt n is equal to the number of clients.

Design mask creation (FedProt step)

Each client has a design matrix X^i with dimensions $m^i \times v$ and an intensity matrix Y^i with dimensions $n \times m^i$, where n is the number of protein groups shared between clients, v is the number of variables in the design, and m^i is the number of samples. The binary mask matrix D has the number of rows equal to protein groups n, and columns corresponding to columns in the design matrix (see **Supplementary Figure 20**)

In the first step, local masks D^i are generated by each client (**Supplementary Figure 20-1**). Clients locally check their data and put "1" into a local mask's cell if either they do not have data for any of the protein groups (all values in $Y^i_{p,\cdot}$ are NA) or they have only "0" in the design for a column (all values in $X^i_{\cdot,s}$ are 0):

$$\mathrm{d}^i_{p,s} = \begin{cases} 1 & \text{if } \forall j \; (Y^i_{p,j} \text{ is NA}) \; \vee \; \forall j \; (X^i_{j,s} = 0) \\ 0 & \text{otherwise} \end{cases},$$

where $d^i_{p,s}$ is an element of the local mask D^i for the i-th client, $p = 1, \ldots, n$ indexes the protein groups, $s = 1, \ldots, v$ indexes the variables in the design, and $j = 1, \ldots, m_i$ indexes the samples.

After local clients' masks are aggregated, the Coordinator checks the number of "1" for each column-row combination and if this number is equal to the number of clients (k), then the element $d_{p,s}$ of the global mask D has "1" (True) for this value (**Supplementary Figure 20-2**). Mathematically, this is represented as:

$$\mathrm{d}_{p,s} = \begin{cases} 1 & \text{if } \sum_{i=1}^{k} \mathrm{d}^i_{p,s} = k \\ 0 & \text{otherwise} \end{cases}.$$

This aggregated mask is sent back to the clients, and only the reference client updates it (**Supplementary Figure 20-3**). If a reference client doesn't have data for a specific protein (row), the last present client becomes the reference instead, and is excluded from the design by replacing "0" (False) with "1" (True) in the mask. This ensures that if all data for a protein group are NA, the last zero within the relevant batch (TMT batch, cohort) columns is set to one, ensuring the proper exclusion of the protein group from the design and subsequent calculations.

Aggregation is performed similarly to step 2 (**Supplementary Figure 20-4**):

$$\mathrm{d}_{p,s} = \begin{cases} 1 & \text{if } \sum_{i=1}^{k} \mathrm{d}^i_{p,s} > 0, \\ 0 & \text{otherwise} \end{cases}.$$

Protein group (row) True columns will be excluded from the design and subsequent calculations. The mask is created only for binary variables in the design (cohorts, target classes). If categorical variables need to be analyzed, we suggest transforming them to binary before analysis.

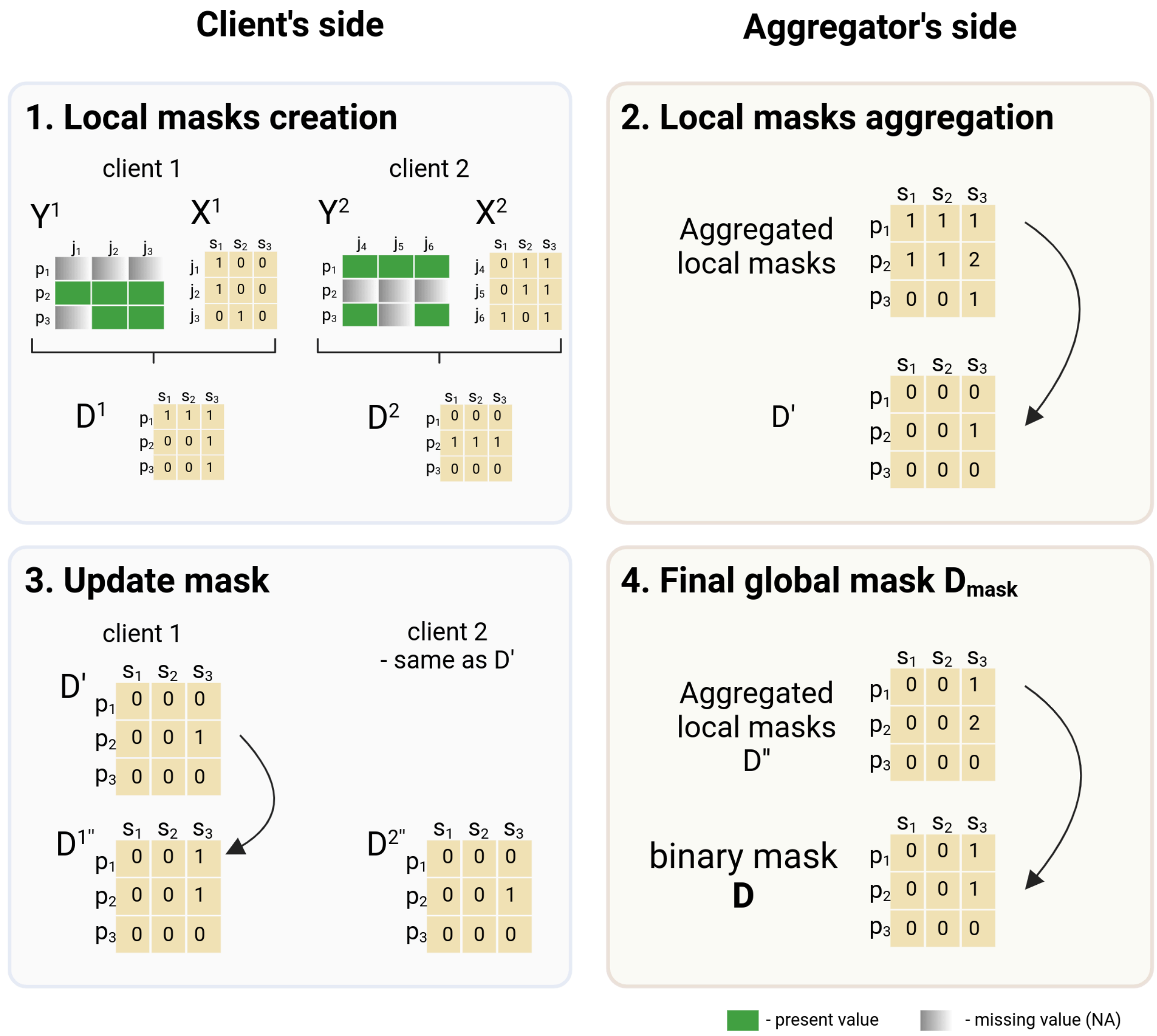

Supplementary Figure 20. Visual representation of the binary mask D creation process in FedProt.

To facilitate visualization, the example is drawn for 2 clients. However, the minimum number of clients in FedProt is 3. The formulas and descriptions are given in the text. The figure was created with BioRender.com.

Supplementary References

1. Čuklina, J. *et al.* Diagnostics and correction of batch effects in large-scale proteomic studies: a tutorial. *Mol. Syst. Biol.* **17**, e10240 (2021).
2. Johnson, W. E., Li, C. & Rabinovic, A. Adjusting batch effects in microarray expression data using empirical Bayes methods. *Biostatistics* **8**, 118–127 (2007).
3. Voß, H. *et al.* HarmonizR enables data harmonization across independent proteomic datasets with appropriate handling of missing values. *Nat. Commun.* **13**, 3523 (2022).
4. Ritchie, M. E. *et al.* limma powers differential expression analyses for RNA-sequencing and microarray studies. *Nucleic Acids Res.* **43**, e47 (2015).
5. Zolotareva, O. *et al.* Flimma: a federated and privacy-aware tool for differential gene expression analysis. *Genome Biol.* **22**, 338 (2021).
6. Nygaard, V., Rødland, E. A. & Hovig, E. Methods that remove batch effects while retaining group differences may lead to exaggerated confidence in downstream analyses. *Biostat. Oxf. Engl.* **17**, 29–39 (2016).
7. Demichev, V. vdemichev/DiaNN: DIA-NN - a universal automated software suite for DIA proteomics data analysis. https://github.com/vdemichev/DiaNN (2025).
8. Adamowicz, K. *et al.* Proteomic meta-study harmonization, mechanotyping and drug repurposing candidate prediction with ProHarMeD. *Npj Syst. Biol. Appl.* **9**, (2023).
9. Clark, D. J. *et al.* Integrated Proteogenomic Characterization of Clear Cell Renal Cell Carcinoma. *Cell* **179**, 964-983.e31 (2019).
10. Zhang, H. *et al.* Proteogenomics of clear cell renal cell carcinoma response to tyrosine kinase inhibitor. *Nat. Commun.* **14**, 4274 (2023).
11. Qu, Y. *et al.* A proteogenomic analysis of clear cell renal cell carcinoma in a Chinese population. *Nat. Commun.* **13**, 2052 (2022).
12. Eldjarn, G. H. *et al.* Large-scale plasma proteomics comparisons through genetics and disease associations. *Nature* **622**, 348–358 (2023).
13. Karr, A. F., Lin, X., Sanil, A. P. & Reiter, J. P. Secure Regression on Distributed Databases. *J. Comput. Graph. Stat.* **14**, 263–279 (2005).
14. Burankova, Y. Freddsle/FedProt: FedProt_evaluation. Zenodo https://doi.org/10.5281/zenodo.15370419 (2025).
15. Fisher, R. A. Statistical Methods for Research Workers. in (Oliver and Boyd, Edinburgh, UK, 1925).
16. Stouffer, S. A., Suchman, E. A., DeVinney, L. C., Star, S. A. & Williams Jr, R. M. *The American Soldier: Adjustment during Army Life.(Studies in Social Psychology in World War Ii)*. vol. 1 (Princeton Univ Press, 1949).
17. Toro-Domínguez, D. *et al.* A survey of gene expression meta-analysis: methods and applications. *Brief. Bioinform.* **22**, 1694–1705 (2021).
18. Tseng, G. C., Ghosh, D. & Feingold, E. Comprehensive literature review and statistical considerations for microarray meta-analysis. *Nucleic Acids Res.* **40**, 3785–3799 (2012).
19. Kaever, A. *et al.* Meta-Analysis of Pathway Enrichment: Combining Independent and Dependent Omics Data Sets. *PLoS ONE* **9**, e89297 (2014).
20. Whitlock, M. C. Combining probability from independent tests: the weighted Z-method is superior to Fisher's approach. *J. Evol. Biol.* **18**, 1368–1373 (2005).
21. Prada, C., Lima, D. & Nakaya, H. MetaVolcanoR: Gene Expression Meta-analysis

Visualization Tool. Bioconductor version: Release (3.17) https://doi.org/10.18129/B9.bioc.MetaVolcanoR (2023).
22. Wang, X. *et al.* An R package suite for microarray meta-analysis in quality control, differentially expressed gene analysis and pathway enrichment detection. *Bioinformatics* **28**, 2534–2536 (2012).
23. Choi, J. K., Yu, U., Kim, S. & Yoo, O. J. Combining multiple microarray studies and modeling interstudy variation. *Bioinformatics* **19**, i84–i90 (2003).
24. Hong, F. *et al.* RankProd: a bioconductor package for detecting differentially expressed genes in meta-analysis. *Bioinformatics* **22**, 2825–2827 (2006).
25. Wiśniewski, J. R. Filter-Aided Sample Preparation for Proteome Analysis. in *Microbial Proteomics: Methods and Protocols* (ed. Becher, D.) 3–10 (Springer, New York, NY, 2018). doi:10.1007/978-1-4939-8695-8_1.
26. Doellinger, J., Schneider, A., Hoeller, M. & Lasch, P. Sample Preparation by Easy Extraction and Digestion (SPEED) - A Universal, Rapid, and Detergent-free Protocol for Proteomics Based on Acid Extraction. *Mol. Cell. Proteomics* **19**, 209–222 (2020).
27. Abele, M. *et al.* Unified Workflow for the Rapid and In-Depth Characterization of Bacterial Proteomes. *Mol. Cell. Proteomics* **22**, (2023).
28. Rappsilber, J., Mann, M. & Ishihama, Y. Protocol for micro-purification, enrichment, pre-fractionation and storage of peptides for proteomics using StageTips. *Nat. Protoc.* **2**, 1896–1906 (2007).
29. Müller-Reif, J. B. *et al.* A New Parallel High-Pressure Packing System Enables Rapid Multiplexed Production of Capillary Columns. *Mol. Cell. Proteomics* **20**, 100082 (2021).
30. Matschinske, J. *et al.* The FeatureCloud Platform for Federated Learning in Biomedicine: Unified Approach. *J. Med. Internet Res.* **25**, e42621 (2023).
