# Supplementary material for: Privacy-preserving multicenter differential protein abundance analysis with FedProt
Source: Nat Comput Sci. 2025 Jul 11;5(8):675–88. doi: 10.1038/s43588-025-00832-7 (PMC12374843; doi:10.1038/s43588-025-00832-7)
Supplement: Supplementary file 2 — Reporting Summary [file 43588_2025_832_MOESM2_ESM.pdf]

nature portfolio

Corresponding author(s): Yuliya Burankova

Last updated by author(s): Jun 2, 2025

# Reporting Summary

Nature Portfolio wishes to improve the reproducibility of the work that we publish. This form provides structure for consistency and transparency in reporting. For further information on Nature Portfolio policies, see our Editorial Policies and the Editorial Policy Checklist.

## Statistics

For all statistical analyses, confirm that the following items are present in the figure legend, table legend, main text, or Methods section.

| n/a | Confirmed | |
|---|---|---|
| ☐ | ☒ | The exact sample size (*n*) for each experimental group/condition, given as a discrete number and unit of measurement |
| ☐ | ☒ | A statement on whether measurements were taken from distinct samples or whether the same sample was measured repeatedly |
| ☐ | ☒ | The statistical test(s) used AND whether they are one- or two-sided<br>*Only common tests should be described solely by name; describe more complex techniques in the Methods section.* |
| ☐ | ☒ | A description of all covariates tested |
| ☐ | ☒ | A description of any assumptions or corrections, such as tests of normality and adjustment for multiple comparisons |
| ☐ | ☒ | A full description of the statistical parameters including central tendency (e.g. means) or other basic estimates (e.g. regression coefficient) AND variation (e.g. standard deviation) or associated estimates of uncertainty (e.g. confidence intervals) |
| ☐ | ☒ | For null hypothesis testing, the test statistic (e.g. *F*, *t*, *r*) with confidence intervals, effect sizes, degrees of freedom and *P* value noted<br>*Give P values as exact values whenever suitable.* |
| ☒ | ☐ | For Bayesian analysis, information on the choice of priors and Markov chain Monte Carlo settings |
| ☒ | ☐ | For hierarchical and complex designs, identification of the appropriate level for tests and full reporting of outcomes |
| ☒ | ☐ | Estimates of effect sizes (e.g. Cohen's *d*, Pearson's *r*), indicating how they were calculated |

*Our web collection on statistics for biologists contains articles on many of the points above.*

## Software and code

Policy information about availability of computer code

| | |
|---|---|
| Data collection | The raw mass-spectrometry data were collected using the following combinations equipment and the software supplied with it and Evosep One – Exploris 480; nanoElute – timsTOF Pro; Ultimate3000 – Orbitrap Fusion Lumos; EASY-nLC 1200 – Exploris 480; Ultimate3000 – QE-HFX; EASY-nLC 1200 – QExactive HF; Ultimate3000 nano – Exploris 480; EASY-nLC 1200 – Orbitrap Fusion Lumos. |
| Data analysis | MaxQuant v 2.4.2, DIA-NN v 1.8.1 and v.1.8.0, Spectronaut v 17.5 and v. 17.2, Docker v.27.1.2.<br>Python (v.3.11.9) packages: pandas v.2.2.2, numpy v.2.0.0, statsmodels v.0.14.2, scipy v.1.14.0, matplotlib v.3.8.4, seaborn v.0.13.2, scikit-learn v.1.5.0, upsetplot v.0.9.0, plotly v.5.22.0.<br>Python package featurecloud v.0.0.32 and related packages to FeatureCloud app run: rpy2 v.3.5.11.<br>R (v.4.2.0) libraries: DEqMS v1.16.0, limma v3.54.2, diann v1.0.1, RobNorm v0.1.0, invgamma v1.1, RankProd v3.24.0, MetaVolcanoR v1.12.0, metaDE v2.2.3, ggrepel v0.9.3, data.table v1.14.8, gridExtra v2.3, patchwork v1.1.2, reshape2 v1.4.4, matrixStats v1.3.0, tidyverse v2.0.0 (includes ggplot2 v3.4.2, dplyr v1.1.4, purrr v1.0.2, readr v2.1.4, tidyr v1.3.1), PRONE v1.0.4.<br>Code for running the analysis is located in GitHub repo: https://github.com/Freddsle/FedProt/tree/main. |

For manuscripts utilizing custom algorithms or software that are central to the research but not yet described in published literature, software must be made available to editors and reviewers. We strongly encourage code deposition in a community repository (e.g. GitHub). See the Nature Portfolio guidelines for submitting code & software for further information.

# Data

Policy information about availability of data

All manuscripts must include a data availability statement. This statement should provide the following information, where applicable:

- Accession codes, unique identifiers, or web links for publicly available datasets
- A description of any restrictions on data availability
- For clinical datasets or third party data, please ensure that the statement adheres to our policy

The mass spectrometry proteomics data have been deposited to the ProteomeXchange Consortium via the PRIDE partner repository with the dataset identifiers PXD053812 (the bacterial dataset, https://www.ebi.ac.uk/pride/archive/projects/PXD053812) and PXD053560 (the human serum dataset, https://www.ebi.ac.uk/pride/archive/projects/PXD053560). Uniprot human reference proteome version 2023_05 (Uniprot UP000005640, reviewed/Swiss-Prot entries only, 20,418 protein sequences, https://www.uniprot.org/proteomes/UP000005640) and Escherichia coli (strain K12) reference proteome (Uniprot UP000000625, 4448 entries, https://www.uniprot.org/proteomes/UP000000625) were used for the human serum and bacterial datasets, respectively. Three datasets (clear-cell renal cell carcinoma, ccRCC) were used for studying robustness against preprocessing variability from the PRIDE66 repository (https://www.ebi.ac.uk/pride/) with the dataset identifiers: PDC000127, PXD042844, PXD030344.
Results and code to obtain them are available via Zenodo at https://doi.org/10.5281/zenodo.15370419 or at the FedProt repository via GitHub at https://github.com/Freddsle/FedProt (inside the data and evaluation folders). The minimal datasets (bacterial, human serum, and three simulated scenarios used in the main manuscript) can be found in the same repository inside the data folder. Source data for Figure 2 is available with this manuscript.

# Human research participants

Policy information about studies involving human research participants and Sex and Gender in Research.

| | |
|---|---|
| Reporting on sex and gender | Both male (n=31) and female (n=29) samples were included, age 19-79 (Supplementary table 12). The gender was based on self-reporting. We have written consent to share the data. The gender was not included in the study design. No sex or gender analysis have been included. |
| Population characteristics | 60 independent human blood serum samples, comprising 30 from patients with primary FSGS and 30 from healthy controls. Clinical characteristics (control or FSGS) and covariate characteristics are reported in Supplementary Table 12. |
| Recruitment | Samples were taken from the nephrological biobank of the Klinikum Bayreuth. Written consent for anonymized data retrieval and storage was obtained. |
| Ethics oversight | The local ethics committee of the Friedrich-Alexander Universität Erlangen-Nürnberg. |

Note that full information on the approval of the study protocol must also be provided in the manuscript.

# Field-specific reporting

Please select the one below that is the best fit for your research. If you are not sure, read the appropriate sections before making your selection.

☒ Life sciences ☐ Behavioural & social sciences ☐ Ecological, evolutionary & environmental sciences

For a reference copy of the document with all sections, see nature.com/documents/nr-reporting-summary-flat.pdf

# Life sciences study design

All studies must disclose on these points even when the disclosure is negative.

| | |
|---|---|
| Sample size | No formal statistical power analysis was performed as our objective was to compare our method to a central reference, not to discover novel effects. Sample sizes were selected based on data availability and on prior experience with similar datasets. This study used a TMT human plasma dataset (60 samples: 2 conditions, 3 cohorts), an LFQ Escherichia coli dataset (118 samples: 2 conditions, 5 cohorts), and simulated data (600 samples: 2 conditions, 3 cohorts). These sample sizes reflect those commonly used in biomarker discovery studies, ensuring adequate representation of the expected variability. |
| Data exclusions | 1 outlier was excluded in E.coli MS-based dataset after data quality control. As our workflow relied on in silico reference samples, 6 pooled reference samples measured in TMT human serum dataset were not used in the analysis. |
| Replication | We tested our method on a variety of real-word and simulated datasets to ensure reproducibility and robustness of the results. Analysis performed independently on real datasets 3 times (3 multi-center datasets used) and 150 times for simulated datasets (50 runs for each scenario). |
| Randomization | E.coli samples were randomly distributed across 5 research centers. For synthetic data, each data simulation and analysis was repeated 50. times. Human serum dataset samples were blinded and distributed to three studies centers by the clinical partners. |
| Blinding | Human serum dataset samples were blinded and distributed to three studies centers by the clinical partners. Samples were prepared and |

| Blinding | measured by independent researchers in a blinded manner until complete data collection. Patient group allocations were disclosed for data analysis. Revealing the information about samples, e.g. class labels, could not influence on the result of the experiments. |
|---|---|

# Reporting for specific materials, systems and methods

We require information from authors about some types of materials, experimental systems and methods used in many studies. Here, indicate whether each material, system or method listed is relevant to your study. If you are not sure if a list item applies to your research, read the appropriate section before selecting a response.

## Materials & experimental systems

| n/a | Involved in the study |
|---|---|
| ☒ | ☐ Antibodies |
| ☒ | ☐ Eukaryotic cell lines |
| ☒ | ☐ Palaeontology and archaeology |
| ☒ | ☐ Animals and other organisms |
| ☒ | ☐ Clinical data |
| ☒ | ☐ Dual use research of concern |

## Methods

| n/a | Involved in the study |
|---|---|
| ☒ | ☐ ChIP-seq |
| ☒ | ☐ Flow cytometry |
| ☒ | ☐ MRI-based neuroimaging |
